# Integrative analysis of vascular endothelial cell genomic features identifies AIDA as a coronary artery disease candidate gene

**DOI:** 10.1186/s13059-019-1749-5

**Published:** 2019-07-08

**Authors:** Simon Lalonde, Valérie-Anne Codina-Fauteux, Sébastian Méric de Bellefon, Francis Leblanc, Mélissa Beaudoin, Marie-Michelle Simon, Rola Dali, Tony Kwan, Ken Sin Lo, Tomi Pastinen, Guillaume Lettre

**Affiliations:** 1 Montreal Heart Institute, 5000 Belanger street, Montréal, Québec H1T 1C8 Canada; 2 Faculté de Médecine, Université de Montréal, Montréal, Québec H3T 1J4 Canada; 3 McGill University and Genome Québec Innovation Center, Montréal, Québec H3A 0G1 Canada; 4 Center for Pediatric Genomic Medicine (CPGM), Children’s Mercy Kansas City, 2401 Gillham Road, Kansas City, MO 64108 USA

**Keywords:** Vascular endothelium, Endothelial dysfunction, Coronary artery disease, Blood pressure, Hypertension, Genome-wide association study, Hi-C, *AIDA*, CRISPR/Cas9

## Abstract

**Background:**

Genome-wide association studies (GWAS) have identified hundreds of loci associated with coronary artery disease (CAD) and blood pressure (BP) or hypertension. Many of these loci are not linked to traditional risk factors, nor do they include obvious candidate genes, complicating their functional characterization. We hypothesize that many GWAS loci associated with vascular diseases modulate endothelial functions. Endothelial cells play critical roles in regulating vascular homeostasis, such as roles in forming a selective barrier, inflammation, hemostasis, and vascular tone, and endothelial dysfunction is a hallmark of atherosclerosis and hypertension. To test this hypothesis, we generate an integrated map of gene expression, open chromatin region, and 3D interactions in resting and TNFα-treated human endothelial cells.

**Results:**

We show that genetic variants associated with CAD and BP are enriched in open chromatin regions identified in endothelial cells. We identify physical loops by Hi-C and link open chromatin peaks that include CAD or BP SNPs with the promoters of genes expressed in endothelial cells. This analysis highlights 991 combinations of open chromatin regions and gene promoters that map to 38 CAD and 92 BP GWAS loci. We validate one CAD locus, by engineering a deletion of the TNFα-sensitive regulatory element using CRISPR/Cas9 and measure the effect on the expression of the novel CAD candidate gene AIDA.

**Conclusions:**

Our data support an important role played by genetic variants acting in the vascular endothelium to modulate inter-individual risk in CAD and hypertension.

**Electronic supplementary material:**

The online version of this article (10.1186/s13059-019-1749-5) contains supplementary material, which is available to authorized users.

## Background

Genetic discoveries in humans have the potential to unravel novel pathophysiological mechanisms and to pinpoint promising drug targets [1]. However, to meet our expectations, these discoveries ought to be supported by mechanistic studies to decipher how genetic variation modulates disease risk. For genome-wide association study (GWAS) discoveries, the design of such functional experiments is particularly challenging as the vast majority of the associated variants are non-coding. Furthermore, we often ignore in which organ(s) or cell type(s) the variants act. Methods have been developed by which we can quantify the enrichment of GWAS variants within regulatory elements identified by transcriptomic or epigenomic profiling of human samples [2–4]. Although powerful, such methods remain probabilistic and further experiments are required to test their predictions. As a consequence, only a few association signals have been resolved at the molecular level [5–7].

GWAS have identified hundreds of variants associated with coronary artery disease (CAD) [8–10] and blood pressure (BP) or hypertension [11, 12]. Many of these association signals implicate excellent candidate genes and independently confirm some of the biology previously known to influence these diseases, such as the role that blood lipid levels play in CAD risk or the importance of smooth muscle contraction in controlling BP. But for many loci, we ignore how they might contribute to the development of these diseases, either because there are no obvious candidate genes nearby or because the variants are not associated with known risk factors. For instance, for CAD, it is estimated that nearly half of the ~ 140 loci identified by GWAS do not associate with the traditional risk factors (e.g., blood lipids, type 2 diabetes, blood pressure) [13].

Annotation of GWAS discoveries for CAD and BP has revealed an enrichment of associated variants near genes implicated in endothelial functions [9, 12]. Vascular endothelial cells form the inner layer of blood vessels and play a critical role in the etiology of CAD and hypertension. Indeed, healthy endothelial cells form a selective barrier between the blood and the intima for many macromolecules, respond to hemodynamic changes, control the vascular tone, and regulate platelet functions, inflammatory responses, and smooth muscle cell growth and migration [14]. Despite their pathophysiological importance and the noted overlap with GWAS findings, endothelial cells have not been studied extensively to provide further insights into genotype-phenotype associations for CAD and BP/hypertension. Here, we profiled the transcriptome, epigenome, and 3D chromosome conformation of vascular endothelial cells and integrate these results with CAD- and BP-associated genetic variants. Because the effect of genetic variation can be specific to certain pathological states [15], we characterized not only resting endothelial cells, but also cells activated with the inflammatory cytokine tumor necrosis factor-α (TNFα). Finally, we used our datasets to generate mechanistic hypotheses and tested one such prediction at a CAD locus using the CRISPR/Cas9 genome editing system.

## Results

### Transcriptomic and epigenomic changes in endothelial cells upon activation

**Fig. 1:**
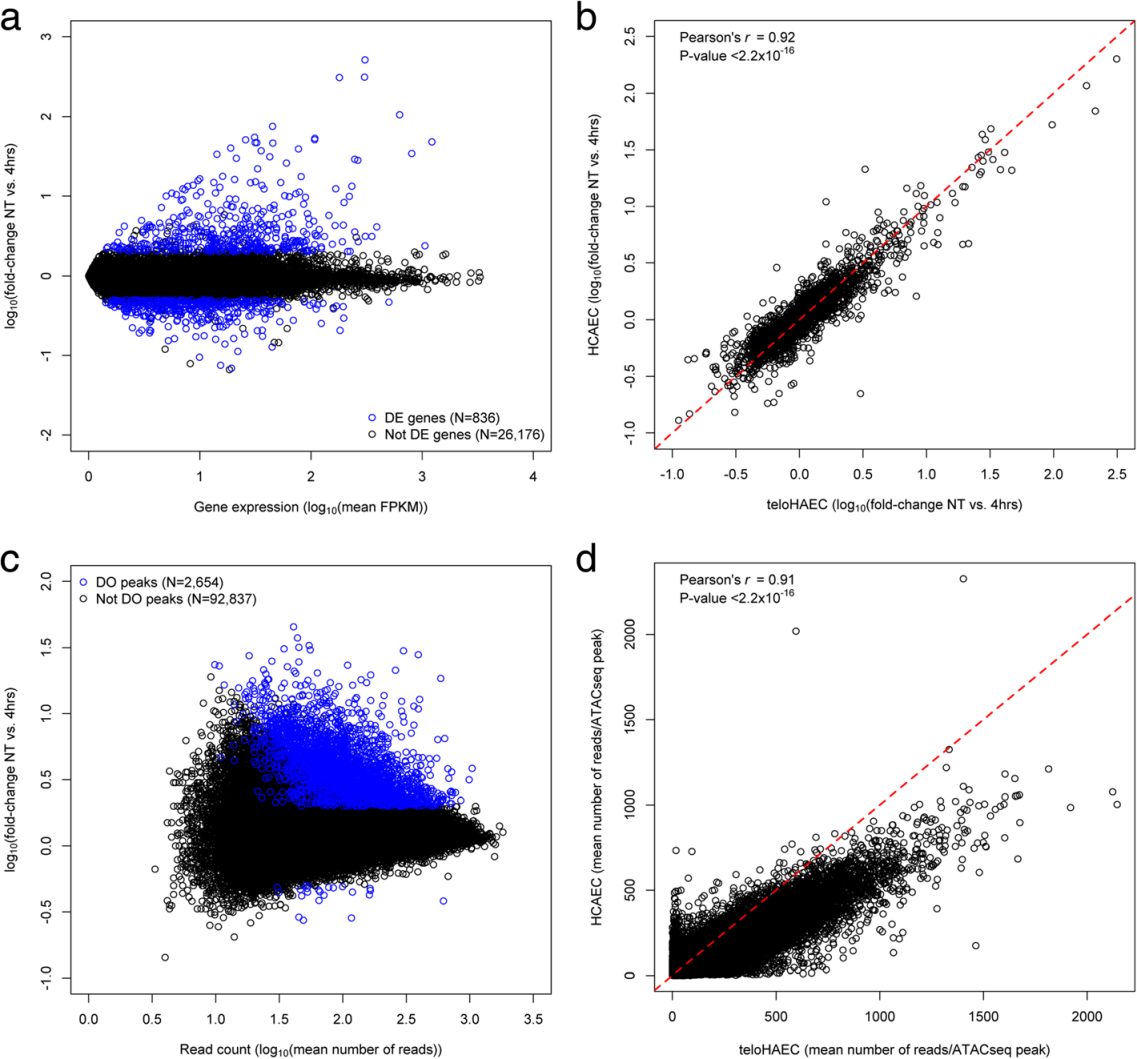
Transcriptomic and epigenomic profiling of teloHAEC. **a** RNAseq of teloHAEC non-treated (NT) or treated with TNFα identified 1316 differentially expressed (DE) genes (FDR < 0.1% and absolute log_10_-fold-change > 0.3) among three comparisons (NT vs. 4 h, NT vs. 24 h, 4 h vs. 24 h). Of these 1316 genes, 836 genes were DE in the NT vs. 4 h comparison. **b** Gene expression fold-change for DE genes are highly correlated between transformed teloHAEC and primary HCAEC. All three comparisons are highly significant (*P* < 2.2 × 10^−16^), but for simplicity we only show the NT vs. 4 h comparison. **c** ATACseq of teloHAEC NT or treated with TNFα identified 95,491 peaks, including 3138 differentially opened (or closed) (DO) peaks (FDR < 0.1% and absolute log_10_-fold-change > 0.3) among three comparisons (NT vs. 4 h, NT vs. 24 h, 4 h vs. 24 h). Of these 3138 peaks, 2654 peaks were DO in the NT vs. 4 h comparison. **d** Open chromatin regions (raw number of reads), identified by ATACseq, are highly correlated between teloHAEC and HCAEC. Results shown are for the 4 h TNFα treatment. Results are consistent for the NT and 24 h timepoints. The distribution falls under the diagonal because the coverage of the ATACseq teloHAEC libraries was higher than the coverage of the HCAEC libraries

To develop a tractable endothelial cellular system to study the molecular mechanisms that contribute to the etiology of vascular diseases such as atherosclerosis and hypertension, we characterized the response of teloHAEC to the potent pro-inflammatory cytokine TNFα. We selected TNFα to activate teloHAEC because it generates a very robust and reproducible inflammatory response [16]. TeloHAEC are immortalized human aortic endothelial cells with many of the cardinal features of endothelial cells (e.g., expression of cell surface marker (CD31/PECAM1), angiogenesis potential, LDL-cholesterol uptake) and a normal karyotype (46,XX; confirmed by cytogenetics and whole-genome DNA sequencing). We treated teloHAEC with TNFα for 4 and 24 h and compared gene expression levels between unstimulated (NT) and stimulated cells by RNA-sequencing (RNAseq). In total when considering all replicates and timepoints, we identified 1316 differentially expressed genes (false discovery rate (FDR) < 0.1% and absolute log_10_ fold-change (|LFC|) > 0.3)(Fig. 1a for the comparison of NT vs. 4 h TNFα treatment, Additional file 1 for all other comparisons, and Additional file 2 for the complete list of differentially expressed genes). Not surprisingly, many of the most upregulated genes are well-known markers of endothelial dysfunction (e.g., *SELE*, *ICAM1*, *SOD2*, *IL8*, *IL1B*) (Additional file 2). Pathway analyses confirmed that most of the transcriptional changes due to TNFα treatment are captured by inflammatory pathways such as TNFα signaling, cytokine-cytokine receptor interaction, and NF-κB signaling (Additional file 3). In parallel, we also treated primary human coronary artery endothelial cells (HCAEC) with TNFα and measured transcript levels by RNAseq. The transcriptional response to TNFα stimulation was highly concordant between immortalized teloHAEC and primary HCAEC in all timepoint comparisons (Pearson’s *r* > 0.6, *P* value < 2.2 × 10^−16^, Fig. 1b), suggesting that teloHAEC represents a good cellular model to study vascular endothelial cell activation.

To correlate changes in gene expression with chromatin activity, we also profiled open chromatin regions by Assay for Transposase-Accessible Chromatin using sequencing (ATACseq) in teloHAEC treated or not with TNFα for 4 or 24 h. By combining data from these different time points, we identified 95,491 ATACseq peaks, including 3138 peaks (3.3%) that are differentially opened or closed (FDR < 0.1% and |LFC|) > 0.3) upon TNFα stimulation (Fig. 1c for the comparison of NT vs. 4 h TNFα treatment, Additional file 1 for all other comparisons, and Additional file 4 for the complete list of differentially opened or closed ATACseq peaks). Although results in Fig. 1c seem to indicate that most ATACseq peaks open upon TNFα treatment, a density analysis of these data points shows that most ATACseq peak LFC are centered at 0 (Additional file 5). As for the transcriptional response, the magnitude of open chromatin regions defined by ATACseq was highly concordant between teloHAEC and HCAEC (Fig. 1d). We employed an in silico footprinting method to determine which transcription factor binding motifs are over-represented within differentially opened teloHAEC ATACseq peaks following TNFα treatment (Additional file 6) [17]. Many of these transcription factors are involved in inflammatory responses (e.g., *JUN*, *FOS*, *NFKB1/2*) (Additional file 7). To further characterize our ATACseq open chromatin dataset, we generated histone H3 lysine 27 acetylation (H3K27ac) data in NT and TNFα-treated teloHAEC using chromatin immunoprecipitation followed by sequencing (ChIPseq). H3K27ac marks highlight regions of active transcription and are found at enhancers and promoters [18]. Within each condition (NT or with TNFα), we found that 70–74% of the ATACseq peaks intersected with H3K27ac peaks.

**Fig. 2:**
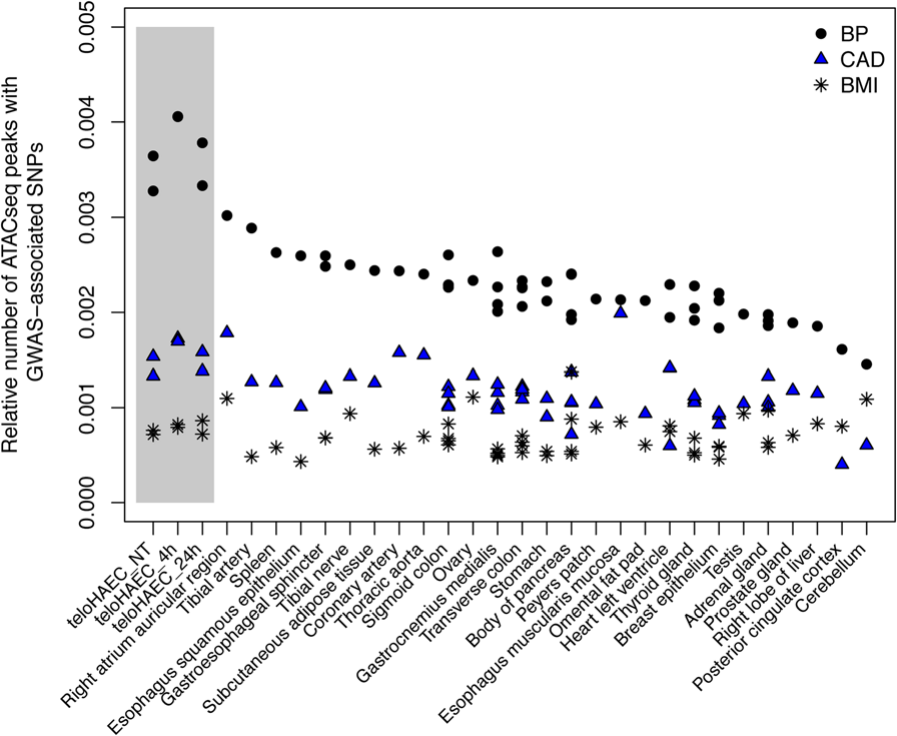
Enrichment of blood pressure (BP) and coronary artery disease (CAD)-associated SNPs in open chromatin regions. We compared overlap in endothelial cells (teloHAEC) and all available tissues from ENCODE. Each biological replicate is identified by a different point. We called all ATACseq peaks with the same bioinformatic pipeline. To account for the different coverage of each ATACseq library, we present the relative fraction of ATACseq peak that overlap with a BP or CAD SNP. We used body mass index (BMI)-associated SNPs as controls, since BMI is not a vascular phenotype. The gray box highlights results generated in this study in non-treated (NT) or TNFα-stimulated (4 or 24 h) teloHAEC

Most genetic variation associated with complex human traits by GWAS is in non-coding regions [19, 20]. To evaluate the relevance of our teloHAEC TNFα-stimulated system to study vascular diseases, we measured the enrichment of single nucleotide polymorphisms (SNPs) associated with CAD or BP in ATACseq peaks identified in teloHAEC. For comparison, we also retrieved ATACseq data from 27 different tissues from the ENCODE Project. For these analyses, we considered CAD- and BP-associated SNPs (as well as their linkage disequilibrium (LD) proxies) obtained from recent large-scale meta-analyses: we tested 175 sentinel (5117 LD proxies) CAD and 357 sentinel (13,970 proxies) BP SNPs [8, 11]. We also used 97 SNPs (3953 proxies) associated with body mass index (BMI) as control genetic variants not associated with a vascular phenotype [21]. The fraction of ATACseq peaks that included CAD-associated SNPs was similar between teloHAEC and coronary arteries, although the enrichment was higher for the esophagus muscularis mucosa and the right atrium auricular region (Fig. 2). For BP-associated SNPs, teloHAEC had the strongest enrichment when compared to all other tested tissues (Fig. 2). Furthermore, the fraction of teloHAEC ATACseq peaks with CAD or BP SNPs was higher than for BMI variants (Fig. 2). Enrichments of CAD- and BP-associated SNPs in ATACseq peaks in teloHAEC were highly significant when controlling for the genome-wide distribution of these peaks, although they were not markedly stronger when focusing on differentially opened/closed ATACseq peaks (Additional file 8) [22]. We obtained highly concordant enrichments when we overlapped CAD and BP SNPs with the location of ChIPseq peaks for the histone mark H3K27ac generated in teloHAEC without or with TNFα (Additional file 8). We identified 263 sentinel or proxy SNPs associated with CAD or BP that map onto a transcription factor binding motif found in a teloHAEC ATACseq peak (Additional file 9).

### 3D chromosomal architecture in endothelial cells

One outstanding challenge in gaining biological insights from GWAS discoveries is to connect variants located in non-coding regulatory elements with their target genes. When cells or tissues from many human donors are profiled, it is possible to use the covariance between open chromatin regions and expression levels of nearby genes to infer that connection. As an alternative solution to link genes and regulatory elements in the context of endothelial dysfunction, we generated genomic contact maps by Hi-C using untreated and TNFα-stimulated (4 h) teloHAEC. For each condition, the contact matrices were highly concordant across biological replicates (Pearson’s correlation *r* > 0.95 for the contact matrices at 10-kb resolution across all replicates), allowing us to combine datasets to increase the signal-to-noise ratios of our analyses.

**Fig. 3:**
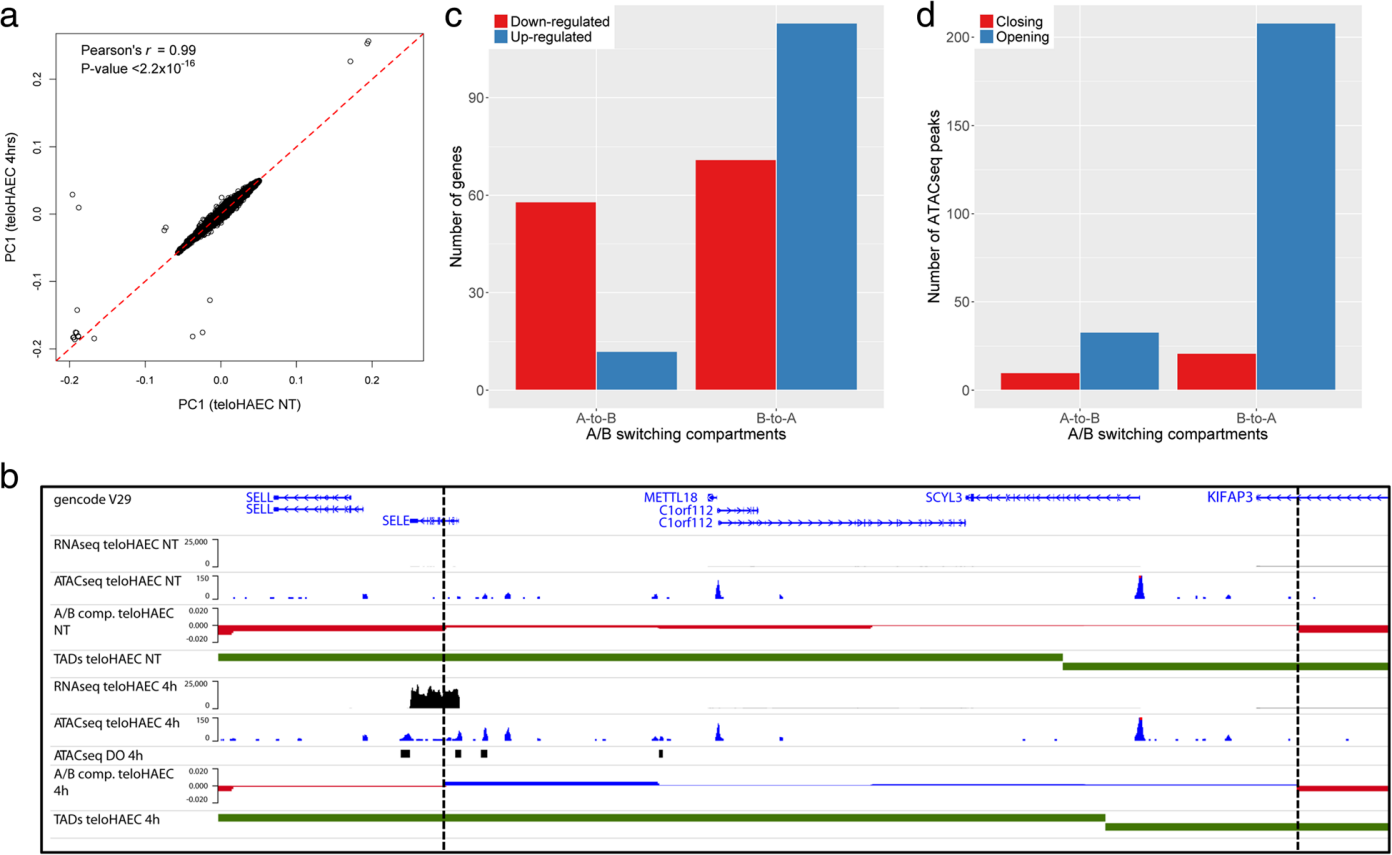
Gene expression and open chromatin regions in switching A/B compartments. **a** Correlation of principal component 1 (PC1) calculated on the Hi-C contact matrices from non-treated (NT) or TNFα-stimulated (4 h) teloHAEC. **b** B-to-A compartment switch at the E-selectin (*SELE*) locus on chromosome 1q24 following TNFα treatment in teloHAEC for 4 h. TNFα treatment strongly induces *SELE* expression (RNAseq NT vs. 4 h) and opens several elements at the locus (ATACseq NT vs. 4 h, differentially opened ATACseq peaks (ATACseq DO 4 h)). The 2 vertical dashed lines indicate the boundaries of a compartment that switch from the repressed B state in NT teloHAEC (red) to the active A state (blue) after 4 h of TNFα treatment. **c** Genes with down-regulated expression after 4 h of TNFα treatment are enriched in active-to-repressed (A-to-B) switching compartments, whereas up-regulated genes are enriched in B-to-A switching compartments. **d** ATACseq peaks that are more opened after TNFα treatment for 4 h are significantly enriched in B-to-A switching compartments

The genome is divided between active and repressed regions, often referred to as A and B compartments [23]. Using principal component analysis on the Hi-C datasets, we identified A and B compartments and compared their distribution genome-wide between NT and TNFα-stimulated teloHAEC. Compartments were highly correlated between conditions, with only 2.1% of the genome that switched following exposure to TNFα (Fig. 3a, b). We compared gene expression (RNAseq) and open chromatin region (ATACseq) changes in teloHAEC (NT vs. 4 h TNFα) with the A/B switching compartments defined by Hi-C. Upregulated genes were enriched in B-to-A compartments (61.4% of genes in B-to-A compartments are upregulated vs. 43.5% of all genes expressed upon TNFα treatment; enrichment = 1.4; *P*_binomial_ = 1.5 × 10^−6^), and downregulated genes were enriched in A-to-B compartments (82.9% of genes in A-to-B compartments are downregulated vs. 56.5% of all genes expressed upon TNFα treatment; enrichment = 1.5; *P*_binomial_ = 4.8 × 10^−6^)(Fig. 3c). ATACseq peaks with higher coverage (opening chromatin) after TNFα stimulation were over-represented in B-to-A switching compartments (90.8% of ATACseq peaks in B-to-A compartments are more open vs. 79.7% of all ATACseq peaks upon TNFα treatment; enrichment = 1.1; *P*_binomial_ = 7.6 × 10^−6^) (Fig. 3d). We could not detect a significant enrichment of ATACseq peaks with lower coverage (closing chromatin) among A-to-B compartments (*P*_binomial_ = 0.57) (Fig. 3d), potentially because there are fewer ATACseq peaks that close after TNFα treatment for 4 h (Fig. 1c).

**Fig. 4:**
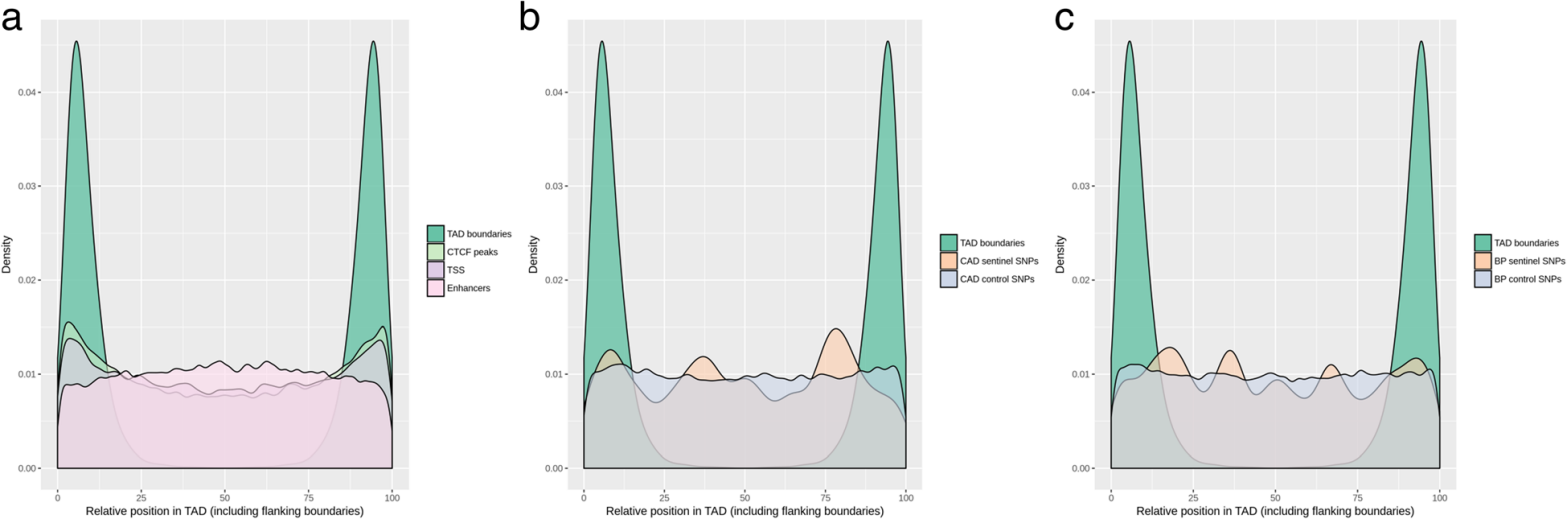
Topologically associated domains (TADs) in teloHAEC endothelial cells treated with TNFα. **a** Because TADs have different sizes across the genome, we normalized them after adding 35 kb on either side to define boundaries. From ENCODE Project data in HUVECs, we retrieved CTCF binding sites from ChIPseq and enhancers defined with histone marks. We used our own RNAseq data in teloHAEC to define transcription start sites (TSS). In **b** and **c**, we map the relative position of coronary artery disease (CAD)- and blood pressure (BP)-associated SNPs into teloHAEC TADs. For comparison, we also added the distribution of relative positions for non-associated, matched (control) SNPs. Similar results were observed for TADs in non-treated teloHAEC (Additional file 10)

Topologically associated domains (TADs) are defined by a high density of interactions between non-adjacent chromosomal regions and represent functional units of genome organization important for gene regulation [24]. We identified 4148 and 4078 TADs in our Hi-C data from NT and TNFα-stimulated teloHAEC, respectively (Fig. 3b). TADs were highly correlated between NT and TNFα-treated endothelial cells, and only 7.7% of TAD boundaries changed upon TNFα treatment. Previous studies have shown that TAD boundaries are enriched with binding motifs for the transcription factor CTCF and the promoters of expressed genes [23, 25]. To validate the TADs that we identified, we retrieved from the ENCODE Project CTCF ChIPseq data from human umbilical vein endothelial cells (HUVEC) and showed that the ENCODE HUVEC CTCF peaks are enriched at teloHAEC TAD boundaries (Fig. 4a and Additional file 10). We also confirmed that transcriptional start sites (TSSs) defined using our teloHAEC RNAseq data were enriched at TAD boundaries (Fig. 4a and Additional file 10). These orthogonal datasets attest to the quality of our Hi-C experiments.

Given the central role that TADs play in the regulation of gene expression, we next asked where within TADs are located ENCODE enhancers predicted by histone marks [18]. In contrast to TSSs, we found that enhancers defined in HUVEC by ENCODE were more uniformly distributed with a slight enrichment in the middle of teloHAEC TADs as opposed to the boundaries (Fig. 4a and Additional file 10). Finally, we mapped CAD- and BP-associated SNPs into TADs and compared their physical distance from the closest TAD boundary with the distance of non-associated matched SNPs. Because of the relatively small number of CAD and BP sentinels SNPs (175 and 357 variants, respectively), the distributions of their position relative to the TAD boundaries were uneven (Fig. 4b, c and Additional file 10). For both CAD and BP, associated SNPs tended to be closer from the nearest TAD boundary than matched SNPs (median distance 75 kb for associated SNPs vs. 103 kb for matched SNPs, empirical *P* values ≤ 0.04), although a larger number of sentinel variants would be needed to provide a definitive answer to this question.

### Linking GWAS SNPs and regulatory elements with genes

We used the Hi-C contact matrices to call loops between regulatory elements that contain CAD- or BP-associated variants and the promoter of genes expressed in teloHAEC. To further refine this list, we applied several criteria: we considered 3D loops supported by ≥ 20 Hi-C reads, we excluded genes that are not expressed or expressed at low levels (bottom 10 percentile) in teloHAEC, and we prioritized open chromatin regions that contain CAD or BP SNPs that are expression quantitative trait loci (eQTL) for the linked genes in the GTEx dataset (*P* value < 0.001) [26]. After filtering, this analysis identified 991 combinations of open chromatin regions and genes linked by physical 3D interactions and eQTL results (Additional files 11, 12, and 13). These combinations map to 38 CAD and 92 BP GWAS loci. The average physical distance between these regulatory elements and gene promoters is 154 ± 158 kb (Additional file 11).

**Fig. 5:**
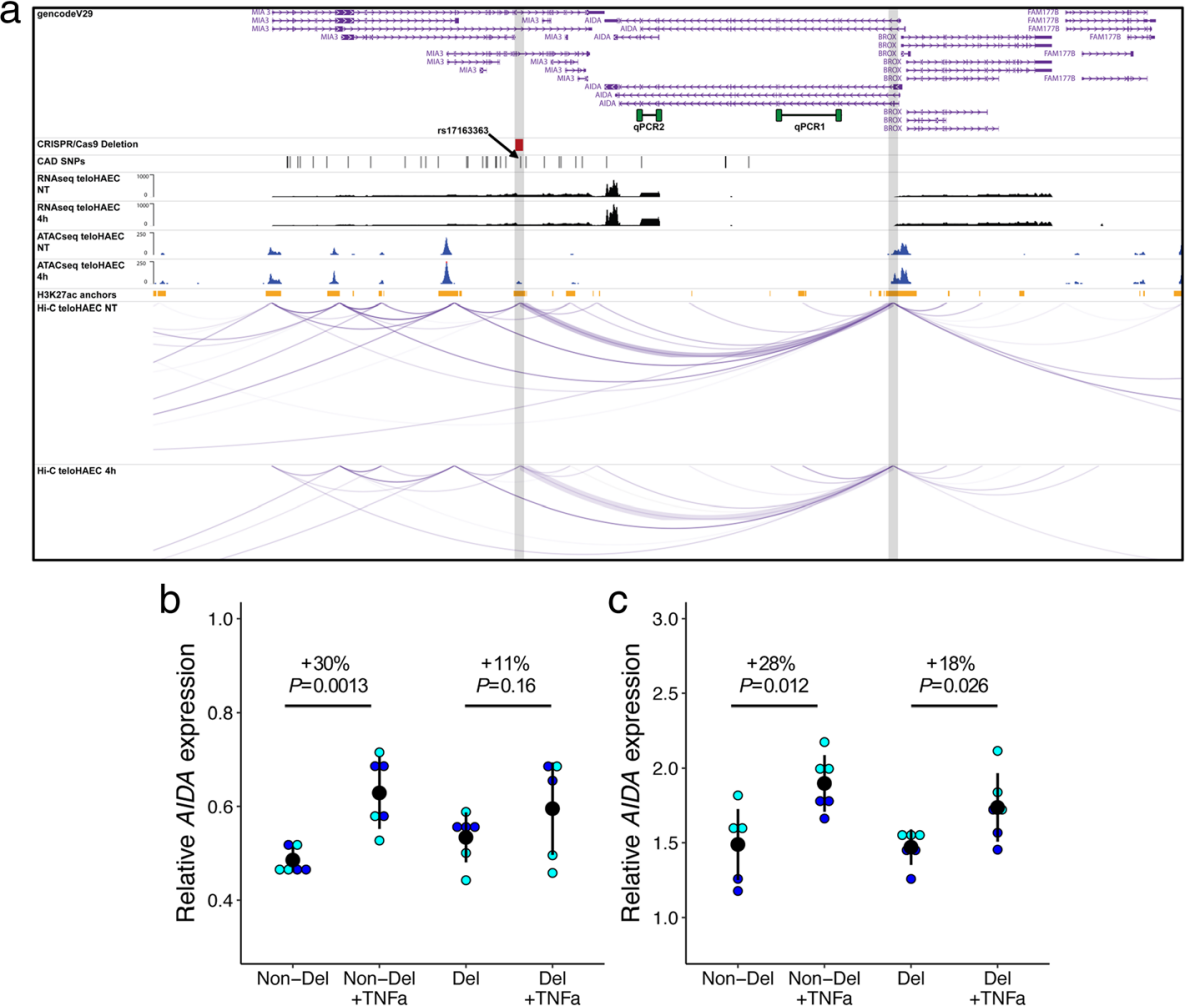
*AIDA* upregulation by TNFα is controlled by a regulatory element that includes one coronary artery disease-associated SNP. **a** Graphical representation of the transcriptomic, epigenomic, and 3D conformation data at the *AIDA* coronary artery disease (CAD)-associated locus. The CRISPR/Cas9 deletion is indicated in red and both qPCR assays are represented in green. We added gray vertical bars to highlight the TNFα-sensitive open chromatin peak (left) and the *AIDA* promoter (right). To improve visualization, we also increased the width of the arcs linking both elements (purple). **b** Relative *AIDA* expression in teloHAEC without or with TNFα treatment for 4 h with qPCR assay #1. Data was obtained from two independent experiments (circles in blue and cyan indicate the different biological replicates). For the non-deleted (Non-Del) and CRISPR/*Cas*9 heterozygote (Del) data points, we pooled data from three independent clones. Mean and standard deviation are plotted (black). For statistical analysis, we used linear regression correcting for batch effects and report two-tailed *P* values. **c** As for **b**, *AIDA* transcript quantification performed with qPCR assay #2

We attempted to validate one of our molecular predictions, focusing on interactions where the open chromatin region and the linked gene are modulated by TNFα treatment and where the variant is a strong eQTL (GTEx *P* value < 1 × 10^−5^). Such additional stringent filtering criteria highlighted two combinations of SNP, open chromatin region and gene at the *AIDA* and *TRAF1* loci (Additional file 11). We selected the *AIDA* locus for further functional characterization because it is associated with CAD; the *TRAF1* SNP is associated with BP (Fig. 5a). Using the CRISPR/Cas9 system in teloHAEC, we engineered a 1022 base pair deletion in the *MIA3* gene that contains a TNFα-sensitive open chromatin element that physically interacts with the promoter of the differentially expressed gene *AIDA*. This deletion encompasses rs17163363, a strong proxy for the sentinel CAD SNP rs67180937 (*r*^2^ = 1 in European populations from the 1000 Genomes Project) that is also an eQTL for *AIDA* in GTEx esophagus muscularis samples (*P*_eQTL_ = 1.4 × 10^−6^). Although we could not retrieve homozygous clones for the deletion, we obtained three independent heterozygotes and tested the induction of *AIDA* expression following TNFα treatment using two different qPCR assays (Fig. 5a and Additional file 14). Whereas TNFα could induce a robust *AIDA* expression response in cells without a deletion (+ 29%, *P*_qPCR1_ = 0.0013 and *P*_qPCR2_ = 0.012), the increase in *AIDA* expression was roughly half that induction level in heterozygous teloHAEC (+ 15%, *P*_qPCR1_ = 0.16 and *P*_qPCR2_ = 0.026), likely because one allele is still functional (Fig. 5b, c). This result is consistent with our model by which this regulatory element—and presumably the genetic variant(s) that it contains—can control the expression of *AIDA* only upon endothelial cell activation.

## Discussion

GWAS have identified hundreds of variants robustly associated with CAD and BP/hypertension. Despite recent efforts, the causal variants, genes, and tissues/cell types remain largely unknown at these loci. In this study, we tested the hypothesis that some of these genetic associations are mediated through the activity of DNA sequence variants that control gene expression upon vascular endothelial cell activation. We profiled the transcriptome (RNAseq) and open-chromatin genome (ATACseq) of resting and TNFα-activated immortalized human aortic endothelial cells (teloHAEC). We focus on these transformed cells in order to develop a system amenable for efficient genome editing experiments, an essential component of any GWAS follow-up program. We confirmed the RNAseq and ATACseq results from teloHAEC in primary human coronary artery endothelial cells. Furthermore, we generated and characterized genome-wide chromosome conformation Hi-C contact matrices from NT and TNFα-treated teloHAEC cells to physically link regulatory elements and expressed genes. By integrating our results with publicly available epigenomic datasets from ENCODE, eQTL results from GTEx, and GWAS discoveries for CAD and BP, we created a dynamic regulatory map of vascular endothelial cells. Through this map, we identified CAD and BP variants that overlap with open chromatin regions which themselves physically interact with often distant gene promoters in a specific cellular inflammation/non-inflammation context (Additional file 11).

To support our results, we tested one prediction by deleting a TNFα-induced ATACseq open chromatin region in teloHAEC using CRISPR/Cas9. In heterozygous clones that carry this ~ 1 kb deletion, the expression of *AIDA* induced by TNFα treatment was strongly hindered (Fig. 5b, c). This is a promising result given that *AIDA* is differentially expressed in teloHAEC following TNFα treatment (NT vs. 4 h, LFC = 0.49, FDR = 5.2 × 10^−19^; Additional file 2) and the *AIDA* promoter interacts with the ATACseq peak as determined by Hi-C (Additional file 11). This locus, defined by the sentinel GWAS variant rs67180937, is associated with CAD and includes 33 other variants in strong LD (*r*^2^ > 0.8 in European populations from the 1000 Genomes Project). Our deletion, however, only encompasses one of these 34 SNPs, rs17163363, which is an eQTL for *AIDA* in GTEx (*P* = 1.4 × 10^−6^). rs17163363 does not overlap perfectly with transcription factor binding motifs, although it is located 14 and 23 base pairs away, respectively, from NKX2-5 and MEF2A binding sites. MEF2 transcription factors have previously been implicated in CAD [27].

Despite several attempts, we failed to identify teloHAEC clones that are homozygous for the ATACseq peak deletion at the *AIDA* locus. This might indicate that baseline expression levels of *AIDA*, *MIA3*, and/or potentially other genes controlled by this regulatory element are essential for teloHAEC cell survival. An extension of this observation is that complete bi-allelic deletion of regulatory elements by CRISPR/Cas9, an approach now routinely attempted to functionally characterize GWAS loci, will often fail or generate negative results that are difficult to interpret. This highlights the importance to develop efficient and high-throughput protocols to combine genome editing and homology-directed repair to precisely replace candidate functional alleles in human cells [28]. Although rs17163363 is the only variant in LD with the CAD sentinel variant rs67180937 within the CRISPR/Cas9 deletion generated at the *AIDA* locus, we cannot conclude that it is causal as other unknown variants in the deleted region could mediate the effect on *AIDA* expression. To address the potential causal role of rs17163363 in CAD, we propose that an allele replacement experiment, potentially mediated by CRISPR/Cas9 homology-directed repair, is needed.

Our results implicate *AIDA* in an inflammatory response that promotes atherosclerosis and CAD. *A*xin *i*nteraction partner and *d*orsalization *a*ntagonist, or *AIDA*, was first identified in a yeast-two-hybrid screen for interaction with the scaffold protein Axin [29]. AIDA homodimerizes but can also physically interact with NFκB inhibitor-α (NFKBIA) and TNFα-induced protein 3 (TNFAIP3) [30], two genes that are highly over-expressed in teloHAEC following TNFα treatment (Additional file 2). In zebrafish, *aida* over-expression in embryos inhibits the dorsalizing activity of Axin by interfering with the activation of the c-Jun N-terminal kinase (JNK) [31]. JNK are multifunctional kinases that are activated by stresses and cytokines, including TNFα, and that can control several cellular stress responses such as apoptosis [32]. In endothelial cells, JNK is also activated in response to pro-inflammatory stimuli [33]. Although it remains speculative, our data leads us to hypothesize that endothelial cell dysfunction mediated by the antagonizing effect of AIDA on JNK contributes to inter-individual variation in CAD risk in humans.

## Conclusions

We anticipate that our integration map of vascular endothelial cell transcriptomic, epigenomic, and 3D conformation datasets, when combined with statistical fine-mapping of GWAS loci, will provide sufficient resolution to pinpoint causal variants and genes implicated in CAD and BP/hypertension. This map will allow further investigation into the roles that endothelial cell dysfunction plays in modulating the risk to develop these important chronic diseases. We illustrated our strategy by characterizing a TNFα-responsive regulatory element that controls the expression of the novel CAD candidate gene *AIDA*. Encouragingly, a recent report identified another CAD-associated regulatory variant of *PLPP3* that resides within a vascular endothelial enhancer activated by shear stress [34], suggesting that many CAD- and BP-associated variants may influence vascular endothelial phenotypes. Finally, our results underscore the critical importance of characterizing both resting and activated cells and lead us to propose a context-dependent, TNFα-induced dysregulation of endothelial *AIDA* expression as a novel candidate mechanism for CAD.

## Methods

### Cell culture

Immortalized human aortic endothelial cells (teloHAEC) (ATCC, CRL-4052) were grown in vascular cell basal media (VCBM) (ATCC, PCS-100-030) supplemented with endothelial cell growth kit-VEGF (ATCC, PCS-100-041) and 200 U/mL penicillin and 200 μg/mL of streptomycin (ThermoFisher, 15140122). Primary human coronary artery endothelial cells (HCAEC) from a single male donor (ATCC, CC-2585) were grown in EGM-2MV (Lonza, CC-3202) supplemented with 200 U/mL penicillin and 200 μg/mL of streptomycin. TeloHAEC and HCAEC were maintained under a 5% CO_2_ atmosphere at 37 °C and subcultured to 90% and 70–85% confluency, respectively. Both cell lines were used below three passages after thawing for all experiments.

### Endothelial dysfunction induction

Endothelial cells were treated with concentrations ranging from 0.1 to 10 ng/mL of TNFα (PeproTech, 300-01A) prepared in culture media for 4 h and 24 h periods. Treatment with 10 ng/mL induced the most substantial endothelial dysfunction related alterations in both teloHAEC and HCAEC without significantly altering cell proliferation and viability. Two independent biological replicates of 10 ng/mL, 4 h only (Hi-C) or 4 and 24 h (RNAseq, ATACseq, ChIPseq) TNFα treatments for each cell line were used for data generation unless stated otherwise. Non-treated (NT) cells grown in parallel were used as control.

### RNA extraction and quantitative PCR

TeloHAEC cells were seeded at 2 × 10^5^ cells per well in 6-well plates, grown for 3 days (refreshed media at day 2) until reaching 95–100% confluency and subjected to TNFα treatment as described above. In order to guarantee the reliability and reproduction of the results, RNA extraction, cDNA synthesis, and qPCR experiments were conducted in accordance to the Minimum Information for Publication of Quantitative Real-Time PCR Experiments (MIQE) guidelines [35]. Total RNA was extracted using RNeasy Plus Mini kit (Qiagen) and analyzed with an RNA 6000 Nano kit (Agilent Technologies) to assess its concentration and integrity on an Agilent 2100 Bioanalyzer. Also, no contamination was found within RNA extracts as assessed by spectrophotometry using Take3 Micro-Volume plates (Biotek) or BioDrop μLite with absorbance ratio of 260/280 nm in a range of 2.0–2.15 for all samples. cDNAs were then generated by reverse transcription from 1 μg of total RNA (with RNA integrity number of 10 for all samples) using 1 U of MultiScribe Reverse Transcriptase, 100 mM dNTPS, 20 U of RNase inhibitor and 1× Random Primers (Applied Biosystems, 4,374,966) in a 20 μL volume reaction. Reverse transcription reaction was carried in three steps: 10 min at 25 °C, 120 min at 37 °C, and 5 min at 85 °C. qPCR reactions were set up with 1.25 μL of cDNA (1/50 dilution based on dynamic range of previously done standard curve for all target genes), 5 μL of Platinum SYBR Green qPCR SuperMix-UDG (ThermoFisher, 11733046), and 3.75 μL of primer pair mix at 0.8 μM each. qPCR reaction for each gene was performed in triplicates and carried out in a CFX384 Touch Real-Time PCR Detection System (Bio-Rad, 1855485) with the following thermal profile: 2 min at 50 °C, 15 min at 95 °C and a three-step cycle of 10 s at 95 °C, 15 s at 55 °C, and 15 s at 72 °C repeated 40 times. Following the amplification process, a melting curve analysis was performed to ensure the specificity of the amplified products. Also, resulting amplification products from previous qPCR standard curve experiments were run on 1% agarose gel and purified prior to Sanger sequencing in order to validate amplification of the desired target. To assert the absence of undesired contamination, qPCR reactions with no template controls for each gene were carried out simultaneously with no fluorescence detected. Cq values corresponding to the number of cycles to reach quantification threshold were determined with the CFX Manager 3.1 (Bio-Rad) software for all genes. Relative expression level for the axin interactor, dorsalization associated (*AIDA*) gene were calculated by the ΔΔCT method [36] normalized with the three reference genes glyceraldehyde 3-phosphate dehydrogenase (*GAPDH*), hypoxanthine phosphoribosyltransferase 1 (*HPRT1*), and TATA-binding protein (*TBP*). Based on geNORM principles for accurate normalization of real-time quantitative RT-PCR data by geometric averaging of multiple internal control genes, a mean *M* value always below 0.35 was generated from the *GAPDH*, *HPRT1*, and *TBP* genes for all qPCR experiments. All primers were obtained from IDT Technologies. The primers sequences are listed in Additional file 15.

### RNAseq and differential gene expression analysis

Stranded cDNA libraries prepared from quality-controlled RNA (see above) were sequenced using Illumina 100-bp paired-ends on a HiSeq 4000 platform, generating 50–60 million reads per condition per biological replicate. Reads were mapped to hg19 using hisat2 (http://ccb.jhu.edu/software/hisat2/index.shtml). Samtools was used to sort the reads and convert to the BAM format. Transcripts were first identified for each sample, and then pooled together using stringtie (http://ccb.jhu.edu/software/stringtie/index.shtml). Transcript abundance was estimated by stringtie, and a fragments per kilobase of transcript per million (FPKM) count table was generated. Differential analysis of gene expression was performed using DESeq2 [37]. All possible comparisons for NT, TNFα 4 h, and 24 h treatments were performed using the analysis of deviance function with default parameters. Genes with a false discovery rate (FDR, Benjamini & Hochberg correction) < 0.1%, and log_10_ fold-change > 0.3 or < − 0.3 in any of the 3 possible comparisons (NT vs. 4 h; NT vs. 24 h; 4 h vs. 24 h) were considered differentially expressed. Corresponding biological replicates output were merged using UCSC BigWig and BigBed tools [38] for visualization purposes in the WashU Epigenome Browser [39].

### Assay for transposase-accessible chromatin with high throughput sequencing (ATACseq)

TeloHAEC and HCAEC cells were seeded at 2 × 10^5^ cells per well in 6-well plates, grown for 3 days (refreshed media at day 2) until reaching 95–100% confluency and subjected to TNFα treatment as described above. Adherent cells were detached using Trypsin-EDTA (ATCC, PSC-999-003) and subsequently neutralized by Trypsin Neutralizing Solution (ATCC, PSC-999-004). Following endothelial cell activation, ATACseq libraries were prepared as previously described [40] with the following specifications and modifications: 5 × 10^4^ cells were spun down at 500 g for 5 min at 4 °C. Whole cell pellets were subjected to a first round of cell membrane lysis using 50 μL of ice-cold hypotonic buffer (0.1% Sodium citrate tribasic dehydrate (Sigma-Aldrich, C8532); 0.1% Triton X-100 (Sigma-Aldrich, X100)) and incubating on ice for 30 min. The hypotonic buffer was removed by centrifugation at 500 g for 5 min at 4 °C, and we subsequently discarded the supernatant. Crude nuclei lysates were prepared by resuspending cells in lysis buffer (10 mM Tris-HCl pH 7.4 (Fisher Scientific, BP-153-1); 10 mM NaCl (Fisher Scientific, BP-358-212); 3 mM MgCl_2_ (Sigma-Aldrich, M8266); 0.1% Igepal CA-630 (Sigma-Aldrich, I8896) and incubating for 30 min on ice. Following the removal of lysis buffer by centrifugation at 500*g* for 5 min at 4 °C, transposase reaction of open chromatin was achieved by resuspending free nuclei in tagmentation mix (22.5 μL Tagment DNA Buffer; 2.5 μL Tagment DNA enzyme; 25 μL H_2_O) (Illumina, FC-121-1030) and incubating at 37 °C for 30 min. Purification of DNA was performed with MinElute (Qiagen, 28004) according to the manufacturer’s protocol. Barcoding and amplification was prepared using Nextera Index Kit (Illumina, FC-121-1011) as previously described [40] with the following thermal profile: 30 s at 98 °C and a three-step cycle of 10 s at 98 °C, 30 s at 63 °C, and 1 min at 72 °C repeated 12 times followed by 5 min at 72 °C. Amplified ATACseq libraries were purified using GeneRead Size Selection Kit (Qiagen, 180514) according to the manufacturer’s protocol. Quality and quantity of final ATACseq libraries were assessed with the High Sensitivity DNA kit (Agilent, 5067-4626) ran on an Agilent 2100 Bioanalyzer. ATACseq libraries were sequenced using Illumina 125-bp paired-ends sequencing on a HiSeq2500 platform with, generating between 38 and 43 million reads per condition per biological replicate.

ATAC library reads were processed through the ATACseq pipeline (https://github.com/kundajelab/atac_dnase_pipelines). Adapters were removed using Cut-adapt. Reads were then mapped to hg19 using Bowtie2. Peak calling from BAM files was performed using MACS2 [41]. To create a “masterBED” peak file across conditions, peak files generated for each condition were merged using the *merge* function from BEDTools [42]. Mean scores from bedGraphs for each individual biological replicate were assigned to masterBED peak files using *intersect* (default parameters) and *merge* (-o mean) and used as input for differential analysis using DESeq2 [37]. All comparisons for NT, TNFα 4 h, and 24 h treatments were performed using the analysis of deviance function with default parameters in DEseq2. ATACseq peaks with a false discovery rate (FDR, Benjamini & Hochberg correction) < 0.1%, and log_10_ fold-change > 0.3 or < − 0.3 in any of the 3 possible comparisons (NT vs. 4 h; NT vs. 24 h; 4 h vs. 24 h) were considered differentially opened or closed. Corresponding biological replicates bedGraphs output from MACS were merged using UCSC BigWig and BigBed tools [36] for visualization purposes in the WashU Epigenome Browser [39]. For in silico footprinting, we used CENTIPEDE with default parameters [17]. For the enrichment analyses of CAD, BP and BMI SNPs in open chromatin regions, we retrieved sentinel variants from published large-scale GWAS [8, 11, 21]. We identified proxy variants in linkage disequilibrium (*r*^2^ > 0.8) using populations of European ancestry from the 1000 Genomes Project [43].

### Chromatin immunoprecipitation of H3K27 acetylation (H3K27ac) combined with high throughput sequencing (ChIPseq)

TeloHAEC were seeded at 1.4 × 10^5^ cells per 100 mm plates (1 plate per condition, 3 independent biological replicates), grown to 90–100% confluency (refreshed media every 2–3 days) and subjected to TNFα treatment as described above. Cells were washed with HBSS (Gibco, 14170161) and fixed in paraformaldehyde (PFA) 1% (Fisher Scientific, 15710) for 10 min at room temperature (RT). PFA was quenched in 134 mM glycine for 5 min at RT. Fixed cells were washed with ice-cold PBS and collected with a cell scraper in ice-cold PBS. Cells were pelleted by centrifugation, washed in ice-cold PBS, and pelleted again before snap freezing in liquid nitrogen. Fixed cells were subject to lysis in 5 mM PIPES-pH 8.5, 85 mM KCl, 1% (*v*/*v*) IGEPAL CA-630, 50 mM NaF, 1 mM PMSF, 1 mM Phenylarsine Oxide, 5 mM Sodium Orthovanadate and protease inhibitor cocktail (Sigma, 04693159001). Nuclei were then lysed in 50 mM Tris-HCl pH 8.0, 10 mM EDTA, 1% (*w*/*v*) SDS, 50 mM NaF, 1 mM PMSF, 1 mM phenylarsine oxide, 5 mM sodium orthovanadate and protease inhibitor cocktail. Chromatin immunoprecipitation was performed as previously described using 3.7 μg of H3K27ac antibody (Diagenode; C15410196) per samples containing ~ 500,000 cells [44]. ChIPseq libraries were sequenced using Illumina 100-bp paired-end read sequencing on a NovaSeq 6000 instrument for approximately 150 million reads per sample. H3K27ac ChIPseq library raw reads were filtered for quality (phred33 ≥ 30) and length (*n* ≥ 32), and adapter sequences were removed using Trimmomatic [45]. Filtered reads were aligned to hg19 using BWA and peaks subsequently called using MACS2 [41] with non-IP input DNA as control. Corresponding bedGraphs output of biological replicates and input controls from MACS were merged using UCSC BigWig and BigBed tools [38] for visualization purposes in the WashU Epigenome Browser [39].

### In situ Hi-C library preparation and analysis

TeloHAEC were seeded at 1.4 × 10^5^ cells per 100 mm plates (4 plates per condition), grown to 90–100% confluency (refreshed media every 2–3 days) and subjected to the 4 h TNFα treatment as described above. In situ Hi-C libraries were prepared as previously described [46] with the following specifications and modifications: approximately 8 × 10^6^ cells per sample were crosslinked, pelleted and washed in ice-cold PBS prior to lysis and chromatin digestion with DpnII. Reverse crosslinking was performed in two subsequent 16 and 2 h incubations with 500 μg of proteinase K prepared at 10 mg/mL in 5 mM Tris-HCl pH 7.5, 50% glycerol, 1 mM CaCl2 for each step. DNA purification was performed using 15 mL MaXtract High Density tubes (Qiagen, 129,065). Pre-NGS Hi-C DNA was quantified and quality-controlled with a DNA 7500 kit (Agilent, 5067-1506) ran on an Agilent 2100 Bioanalyzer. Prior to next-generation DNA sequencing (NGS), DNA extractions for quality control of chromatin integrity, digestion efficacy were performed with the following procedure: 1 volume of Phenol:Chloroform:Isoamyl Alcohol (25:24:1 *v*/*v*) (Invitrogen, 15593031) was added to lysate, vortexed and transferred to pre-spun Phase Lock Gel (VWR, 10847-800) and centrifuged for 5 min at 16,000*g*. The aqueous phase was kept, concentrated by speed-vacuum and subjected to 0.8% agarose gel electrophoresis. Quality-control 3C-PCR of pre-NGS Hi-C libraries was performed in the ENr313 region using 800 ng of template DNA, PfuUltra II Fusion HotStart DNA Polymerase (Agilent, 600672), 400 nM ENr313_DpnII_Anchor1 primer #1, 400 nM ENr313_DpnII_Anchor1_Near primer #2 and 250 μM dNTPs with the following thermal profile: 2 min at 95 °C and a three-step cycle of 30 s at 95 °C, 30 s at 60 °C and 30 s at 72 °C repeated 35 times followed by 8 min at 72 °C [46]. For NGS preparation, between 20 and 40 μg of purified Hi-C DNA was used as starting material for all subsequent steps. Sonication to 200-300 bp fragments was carried in an S2 Focused-ultrasonicator with no alterations to the suggested parameters. Biotin pulldown was performed with 200 μg of Dynabeads MyOne Streptavidin C1 (Invitrogen, 65001) per sample. Production PCR was carried out with 9 cycles of PCR to obtain sufficient quantity for NGS while limiting PCR duplicates. Quality and quantity of final Hi-C libraries were assessed on High Sensitivity DNA kit (Agilent, 5067-4626) ran on an Agilent 2100 Bioanalyzer. Final Hi-C libraries were sequenced using Illumina 100-bp paired-ends sequencing on a Novaseq 6000, generating between 0.72 and 0.88 billion reads per condition per biological replicate.

Hi-C reads were processed using the Juicer pipeline [47]. Hi-C libraries for all biological replicates had reads with the following quality measures: less than 10% below MAPQ threshold of 30 (average of 9.15%), more than 62% intra-chromosomal interactions (average of 68.5%) and less than 26% of inter-chromosomal interactions (average of 20.3%). Correlation between biological replicates was assessed (Pearson’s *r*, 10 kb resolution > 0.95; 50 kb resolution > 0.97; 100 kb resolution > 0.98) before merging to increase statistical power. Contacts maps were normalized with Knight-Ruiz (KR) matrix balancing before all downstream analyses.

### A/B compartments calling and analysis

Per chromosome principal component analysis (PCA) was performed by calling the *eigenvector* function from the Juicer pipeline using 50 kb resolution matrices with KR normalization. Using the R packages TxDb and Sushi, PC1 values were aligned to gene density in 50 kb windows. If needed, the sign of PC1 was adjusted to correlate positive PC1 values with gene-rich regions and negative PC1 with gene-poor regions. Contiguous bins of positive and negative PC1 were labeled as A and B compartments, respectively. Switching from A-to-B and B-to-A compartments upon TNFα treatment was retrieved from the differences in A/B compartments called between NT and TNFα-treated cells. Genes, ATACseq peaks, BP and CAD SNPs mapping to switching compartments were identified using *map* and *merge* functions from BEDTools with default parameters.

### Topologically associated domains (TADs) calling and analysis

TAD calling was performed on teloHAEC (NT and 4 h TNFα). KR normalized sparse matrices of 10 kb resolution were extracted from .hic files by calling the dump function from the Juicer pipeline [47]. TAD calling was performed using the Crane insulation score algorithm [48] Git version eecc2c9, with the default parameters (insulation delta span = 200 kb, insulation square size = 500 kb, insulation mode = “mean,” boundary margin of error = 3, noise threshold = 0.1). TADs that overlap with the centromeres, as well as regions at either end of each chromosome, were excluded from analyses. To determine if a TAD boundary overlapped with a feature (e.g., SNPs, ChIPseq, TSS, enhancer, promoter), we added a 10 kb outward buffer to the boundary coordinates. To determine if TADs were stable or changed following TNFα treatment, we added a 20-kb outward buffer to the boundary coordinates. The physical distance of CAD and BP sentinel SNPs with the closest TAD boundary was compared with the distance of control SNPs matched based on minor allele frequency, gene density, gene proximity and the number of LD proxies using SNPsnap default parameters [49]. To derive empirical *P* values, we considered the median distances to the closest TAD boundary of 100 sets of matched SNPs and compared them to the median distance of the CAD or BP SNPs.

### Loop calling between regulatory regions and promoters

Hi-C reads were processed using the HiC-Pro pipeline (https://github.com/nservant/HiC-Pro). hichipper (https://github.com/aryeelab/hichipper) was used to call loops between promoters and ATACseq peaks that harbor CAD or BP GWAS SNPs. Gene promoters’ coordinates were downloaded from the EPDnew database (https://epd.vital-it.ch/human/human_database.php). The detailed steps used to integrate and combine the GWAS, RNAseq, ATACseq, Hi-C, and GTEx data are provided in Additional file 11.

### CRISPR/Cas9 genome editing

Pairs of guide RNAs (sgRNAs) were designed for each targeted genomic deletion and cloned into the pHKO9 vector under control of the same U6 promoter (Additional file 16). HEK 293 T cells were seeded at 5 × 10^5^ cells/well in 6-well plates for 24 h. Lentivirus were produced by co-transfecting the envelope and packaging plasmids pMD2G and psPAX2 respectively with the dual sgRNA expressing pHKO9 vector in HEK 293 T cells using Lipofectamine 2000 (ThermoFisher, 11,668,027) for 4 h then switched to virus-producing media containing 10 μg/mL of BSA. Viral supernatant was harvested 48 h and 72 h following transfection and filtered through 0.45 μm filters. TeloHAEC cells stably expressing an active Cas9 protein were seeded at 2 × 10^5^ cells/well in 6-well plates and later infected with the virus preparation and media containing 0.7 μg/mL of polybrene (Sigma, H9268). Selection with 200 μg/mL of G418 (Fisher, MT30234CR) was started 48 h post-infection. Antibiotic selective pressure was maintained for 5–6 days or until non-infected cells were dead. Sub-populations of 50 cells were derived and screened via PCR using primers surrounding the expected deletion (out-out PCR) (Additional file 16). Clonal cell lines were then derived from a PCR-positive deletion sub-population. Another round of out-out PCR was performed on these select clonal cell lines and PCR products were purified and cloned into pDrive cloning vector system (Qiagen, 231122) or into the pUC19 vector using In-Fusion HD cloning system (Takara, 638,909). Genotypes of all possible alleles were confirmed by gel electrophoresis (Additional file 14) and Sanger sequencing. Select genome reengineered clones were then seeded at 9 × 10^4^ cells per well in 12-well plates, grown to 90–100% confluency (refreshed media every 2–3 days) and subjected to a 4-h TNFα treatment as described above. Total RNA was then extracted, quantified, quality controlled and reverse transcribed as described above. qPCR was performed for the target gene anchored at the receiving end of the chromatin loop with 2 different primer pairs capturing exons in either 5′ or 3′ of *AIDA*.

## Additional files

Additional file 1: Comparison of RNA-sequencing (A and B) and ATAC-sequencing (C and D) results in teloHAEC non-treated (NT) or treated with TNFα for 4 h or 24 h. (DOCX 377 kb)

Additional file 2: RNA-sequencing results of teloHAEC NT or treated with TNFalpha for 4 or 24 h. (XLSX 216 kb)

Additional file 3: Biological pathways enriched for genes that are differentially expressed in teloHAEC upon TNFα treatment. (XLSX 14 kb)

Additional file 4: ATAC-sequencing results of teloHAEC NT or treated with TNFα for 4 or 24 h. (XLSX 381 kb)

Additional file 5: Log10-fold-change (LFC) of ATACseq peaks comparing non-treated vs. 4 h TNFα-treated teloHAEC. (DOCX 315 kb)

Additional file 6: Transcription factor binding motifs that are represented in ATACseq peaks profiled in NT or TNFα-treated teloHAEC. (XLSX 16 kb)

Additional file 7: Biological pathways that are enriched for transcription factors that bind within ATACseq peaks profiled in NT and TNFα treated teloHAEC. (XLSX 14 kb)

Additional file 8: Enrichment of coronary artery disease (CAD)- and blood pressure (BP)-associated SNPs (or their linkage disequilibrium proxies) in teloHAEC ATACseq or H3K27ac ChIPseq peaks. (XLSX 10 kb)

Additional file 9: Transcription factor (TF) binding motifs located in ATACseq peaks that overlap with SNPs associated with coronary artery disease (CAD) or blood pressure (BP). (XLSX 61 kb)

Additional file 10: Topologically associated domains (TADs) in unstimulated teloHAEC endothelial cells. (DOCX 854 kb)

Additional file 11: Hi-C loops between open chromatin regions and gene promoters in teloHAEC. The regulatory elements were defined using ATACseq data. (XLSX 187 kb)

Additional file 12 Definition of the headers used in Additional file 11. (XLSX 9 kb)

Additional file 13: Detailed steps to generate and integrate the GWAS SNP, RNAseq, ATACseq, eQTL, and HiC data. (XLSX 9 kb)

Additional file 14: Validation of the CRISPR/Cas9-induced deletion at the *AIDA* locus (DOCX 790 kb)

Additional file 15: Primers sequences for quantitative PCR. (XLSX 10 kb)

Additional file 16: Primers used to generate indels in teloHAEC. (XLSX 8 kb)

Additional file 17: Review history. (DOCX 323 kb)

## Data Availability

The datasets generated and/or analyzed during the current study are available in the NCBI’s Gene Expression Omnibus (GEO) repository (GSE126200) [50].

## Abbreviations

ATACseq: Assay for transposase-accessible chromatin with high-throughput sequencing
BMI: Body mass index
BP: Blood pressure
CAD: Coronary artery disease
ChIPseq: Chromatin immunoprecipitation with high-throughput sequencing
DE: Differentially expressed
DO: Differentially opened and/or closed
eQTL: Expression quantitative trait locus
FDR: False discovery rate
GWAS: Genome-wide association study
H3K27ac: Acetylation of lysine-27 on histone 3
HCAEC: Human coronary artery endothelial cells
Hi-C: Chromatin conformation capture with high-throughput sequencing
HUVEC: Human umbilical vein endothelial cells
LD: Linkage disequilibrium
LFC: Log_10_ fold-change
NT: Non-treated
RNAseq: Ribonucleic acid sequencing
SNP: Single nucleotide polymorphism
TAD: Topologically associated domain
teloHAEC: Immortalized human aortic endothelial cells
TNFα: Tumor necrosis factor-α
TSS: Transcription start site
