# Supplementary material for: Integrative analysis of vascular endothelial cell genomic features identifies AIDA as a coronary artery disease candidate gene
Source: Genome Biol. 2019 Jul 8;20:133. doi: 10.1186/s13059-019-1749-5 (PMC6613242; doi:10.1186/s13059-019-1749-5)
Supplement: Supplementary file 1 — Comparison of RNA-sequencing (A and B) and ATAC-sequencing (C and D) results in teloHAEC non-treated (NT) or treated with TNFα for 4 h or 24 h. (DOCX 377 kb) [file 13059_2019_1749_MOESM1_ESM.docx]

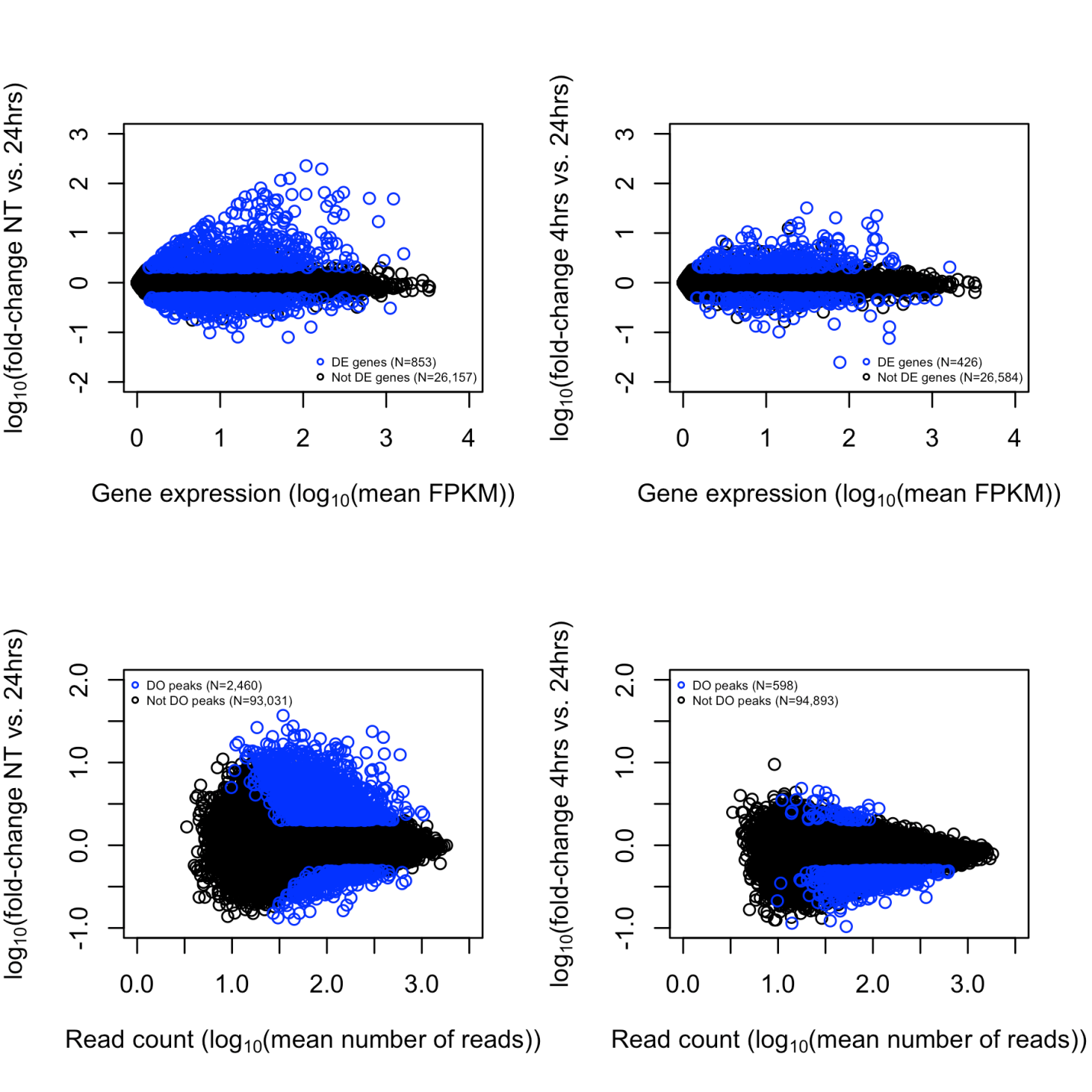


**D**

**C**

**B**

**A**

**Additional file 1. Comparison of RNA-sequencing (A and B) and ATAC-sequencing (C and D) results in teloHAEC non-treated (NT) or treated with TNFα for 4 hours or 24 hours.** (**A**) Log10-fold-change (LFC) in gene expression between NT and 24 hours-TNFα-treated teloHAEC. 853 genes have a false discovery rate (FDR) <0.1% and an absolute LFC >0.3 between these 2 timepoints. (**B**) LFC in gene expression between 4 hours- and 24 hours-TNFα-treated teloHAEC. 426 genes have a FDR <0.1% and an absolute LFC >0.3 between these 2 timepoints. (**C**) LFC in ATACseq peak read counts between NT and 24 hours-TNFα-treated teloHAEC. 2,460 peaks have a FDR <0.1% and an absolute LFC >0.3 between these 2 timepoints. (**D**) LFC in ATACseq peak read counts between 4 hours- and 24 hours-TNFα-treated teloHAEC. 598 peaks have a FDR <0.1% and an absolute LFC >0.3 between these 2 timepoints.
