# Supplementary material for: Integrative analysis of vascular endothelial cell genomic features identifies AIDA as a coronary artery disease candidate gene
Source: Genome Biol. 2019 Jul 8;20:133. doi: 10.1186/s13059-019-1749-5 (PMC6613242; doi:10.1186/s13059-019-1749-5)
Supplement: Supplementary file 5 — Log10-fold-change (LFC) of ATACseq peaks comparing non-treated vs. 4 h TNFα-treated teloHAEC. (DOCX 315 kb) [file 13059_2019_1749_MOESM5_ESM.docx]

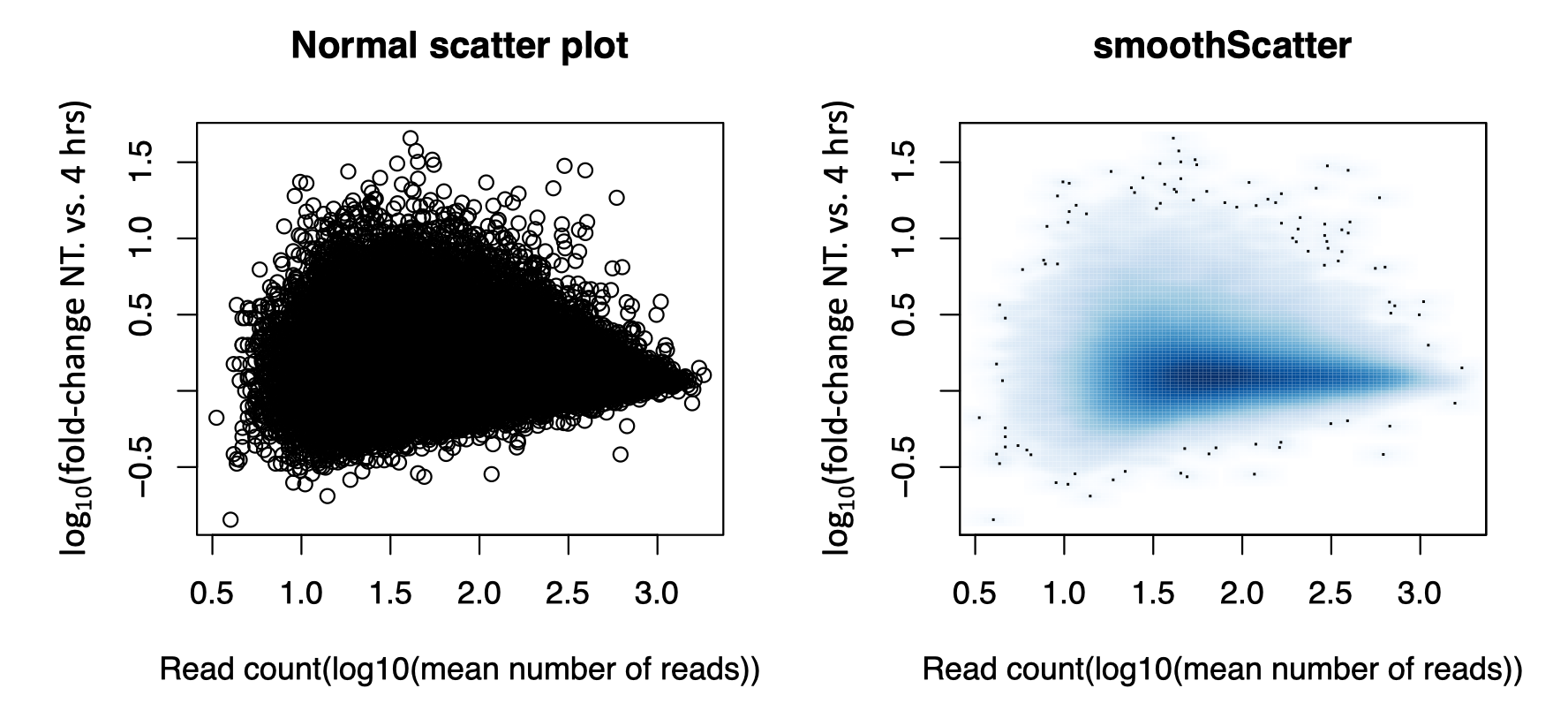


**Additional file 5. Log10-fold-change (LFC) of ATAC-seq peaks comparing non-treated vs. 4 hours TNFα-treated teloHAEC.** The smoothScatter plot shows the same data than on the right, but uses shades of blue to represent data density (darker blue means higher density). Most ATACseq peaks have a LFC that is centered at 0.
