# Supplementary material for: Integrative analysis of vascular endothelial cell genomic features identifies AIDA as a coronary artery disease candidate gene
Source: Genome Biol. 2019 Jul 8;20:133. doi: 10.1186/s13059-019-1749-5 (PMC6613242; doi:10.1186/s13059-019-1749-5)
Supplement: Supplementary file 10 — Topologically associated domains (TADs) in unstimulated teloHAEC endothelial cells. (DOCX 854 kb) [file 13059_2019_1749_MOESM10_ESM.docx]

**Additional file 10. Topologically associated domains (TADs) in unstimulated teloHAEC endothelial cells.** (**A**) Because TADs have different sizes across the genome, we normalized them after adding 35 kb on either side to define boundaries. From ENCODE Project data in HUVECs, we retrieved CTCF binding sites and enhancers defined with histone marks from ChIPseq peaks. We used our own RNAseq data in teloHAEC to define transcription start sites (TSSs). In **B** and **C**, we map the relative position of coronary artery disease (CAD)- and blood pressure (BP)-associated SNPs into teloHAEC TADs. For comparison, we also added the distribution of relative positions for non-associated, matched (control) SNPs. See Methods for details.
