# Supplementary material for: Integrative analysis of vascular endothelial cell genomic features identifies AIDA as a coronary artery disease candidate gene
Source: Genome Biol. 2019 Jul 8;20:133. doi: 10.1186/s13059-019-1749-5 (PMC6613242; doi:10.1186/s13059-019-1749-5)
Supplement: Supplementary file 14 — Validation of the CRISPR/Cas9-induced deletion at the AIDA locus (DOCX 790 kb) [file 13059_2019_1749_MOESM14_ESM.docx]

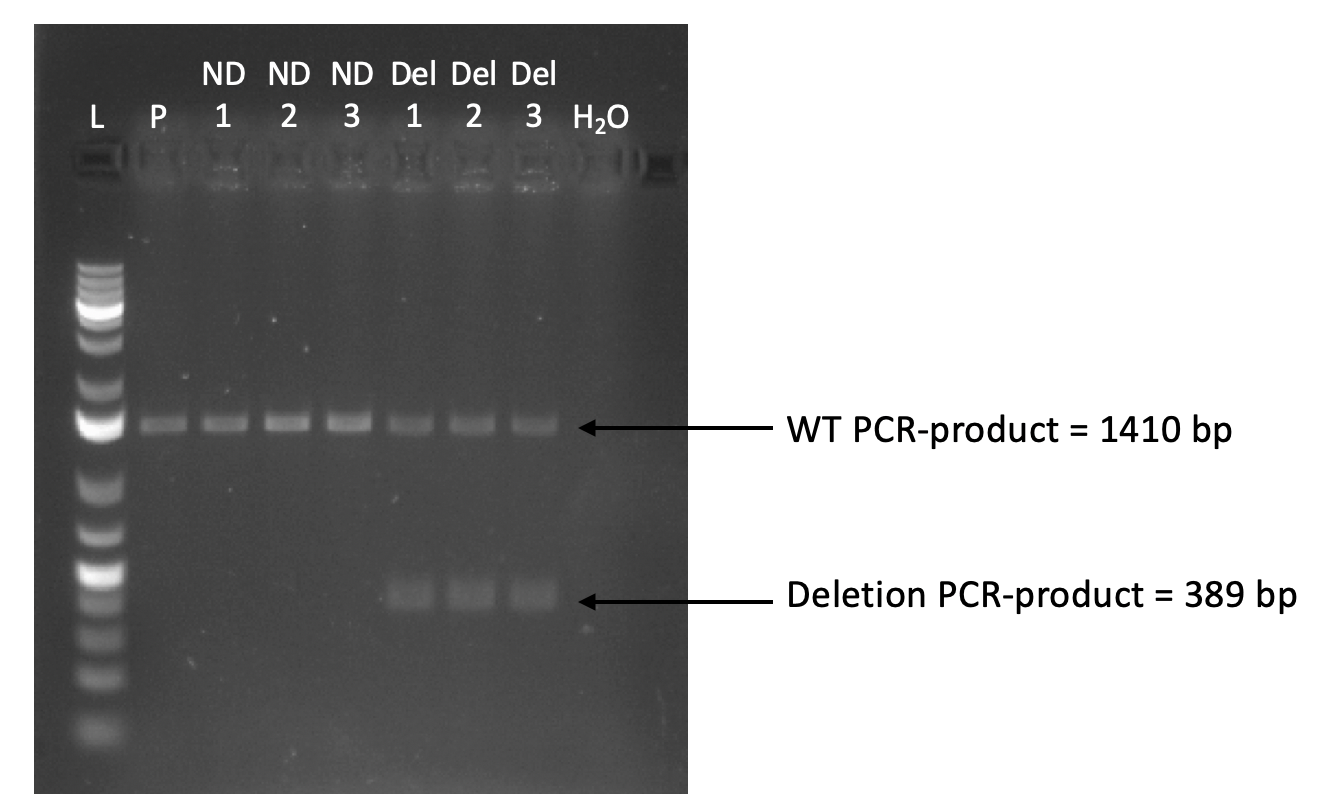


**Additional file 14. Validation of the CRISPR/Cas9-induced deletion at the *AIDA* locus.** Using PCR primers that map outside of the deletion, we amplified the locus in the parental teloHAEC cell line (P), in three independent control clones without a deletion (ND1-3), and in three clones that are heterozygous for the deletion (Del1-3). Cells without the deletion yield a single 1,410-bp PCR product, whereas heterozygous clones yield two bands: the normal (WT) allele at 1,410-bp and the deleted allele at 389-bp. L, 1 kb molecular ladder; H_2_O, negative control without template DNA.
