# Supplementary material for: Integrative analysis of vascular endothelial cell genomic features identifies AIDA as a coronary artery disease candidate gene
Source: Genome Biol. 2019 Jul 8;20:133. doi: 10.1186/s13059-019-1749-5 (PMC6613242; doi:10.1186/s13059-019-1749-5)
Supplement: Supplementary file 17 — Review history. (DOCX 323 kb) [file 13059_2019_1749_MOESM17_ESM.docx]

**First round of review**

**Reviewer 1**

Lalonde, Codina-Fauteux and colleagues Describe genomic data sets for endothelial cells as a resource for interpretation of genetic studies such as those for coronary artery disease. They generated RNA-seq, ATAC-seq, Hi-C and histone mark ChIP-seq data in resting and inflammation-stimulated aortic endothelial cells from a diploid immortalized cell line, teloHAEC and showed concordance with primary aortic endothelial cells. Integrative analyses linked thousands of regulatory elements to promoters and demonstrated that TNF treatment can lead to hundreds of differentially accessible chromatin sites corresponding to changes in gene expression. CRISPR-based heterozygous deletion of one regulatory element show evidence that the element may affect expression of the AIDA gene. 

The data sets created here provide new genomic characterization of endothelial cells in a resting versus a perturbed state. Such perturbation experiments are relatively new, and each example will interest a broad audience in genomics for the approaches and results of perturbation and integrative analyses.

Comments about methods and conclusions:
1. The authors use HiC data to connect regulatory elements to target genes. The links would be more robust if the strength of ATAC-seq signals and RNA-seq expression levels were shown to be correlated (e.g. PMID 30858613)
2. The differential gene expression analyses are described in the text as (linear) absolute fold change, but the plots (Figure 1) and 'Additional file' data are shown in the more typical log fold change. Reporting the number of differentially expressed genes based on the linear threshold seems to over-state the magnitude of differences observed. The text units should match the units in figures and tables. 
3. Figures 1A and 1C show differentially expressed genes and differentially accessible peaks at 4 hours, but the referring text (page 4, lines 18 and 36) reports larger numbers. Which time point comparisons were used to count differential genes and peaks (all of 0-4, 4-24, 0-24?) or was an integrative analysis used? Do the gene and peak differences between timepoints overlap appropriately to justify the choice to combine the timepoints? The manuscript would be more valuable if the rationale for those choices was also explained/justified.
4. The differential accessibility analyses (Figure 1C) appear to show an overall shift of peaks to more accessible after stimulation even among peaks considered not differentially accessible (shown in black), whereas the gene expression appears to be more equally increased versus decreased. Could the shift represent technical differences in ATAC-seq library preparation or analysis? What quality control was performed to determine whether systematic technical differences exist between time points? Alternatively, does the plot just appear to highlight the shifted peaks due to overlaying thousands of points, most of which are centered at log fold change of 0? If the latter, then the authors might consider showing these figures with density plots such as 'smoothScatter' in R ggplot2. 
5. Methods and the threshold used to define linkage disequilibrium proxies of GWAS variants are missing.
6. Figures 4B and 4C show an unusual wavy pattern of position of CAD sentinel SNPs relative to TAD boundaries. The waviness suggests that the results are driven by a very small number of loci or a non-random selection of 'matched' SNPs, affecting the conclusion of enrichment near TAD boundaries. The basis for the waves and impact on the conclusions needs to be described further or the conclusions tempered.
7. Methods for the integrative analysis with eQTL data from GTEx need to be described. Are the reported links to gene using these eQTL data based only on a nominally significant p-value in GTEx or are these loci for which the GWAS and eQTL SNPs have been determined to be colocalized? GTEx has reported that most GWAS variants that show nominal association with expression level of a gene are not colocalized, so most of these 3517 links to genes may be false positives. Here, eQTLs were just used to prioritize among the presumably many more links based on HiC data, so the set can still be used, but the limitation should be noted. The relationship between CAD SNPs and the eQTL at AIDA should be described more explicitly.
8. Further, the strategy applied by the 'in house scripts' (page 15 line 40) used to combine and integrate SNP, ChIP-seq, ATAC-seq, HiC, RNA-seq and GTEx data need to be further described.
9. The accounting of 'combinations' of regulatory elements and promoters would be more informative for validation purposes if it also counted the total number of GWAS loci at which these elements and genes were located.
10. Evidence that the CRISPR clones were heterozygous should be provided.
11. The results described and shown in Figure 5 should more clearly indicate that the 6 points are not independent replicates, as only three heterozygous clonal lines were generated. The methods should better explain how these experiments were performed; were replicate wells of cells stimulated or were replicates performed at the qPCR stage? The independence of the points influences whether the statistics based on 6 points are fair. Given the relatively large variance observed in these assays, the independence of values is especially important and affects conclusions in the Results and Discussion ('could not be induced' page 8 line 30). 
12. The conclusion (Results, page 7, line 4) that the variant located within the element presumably affects expression of AIDA is speculative, as no data specific to the variant is shown. This speculation could be included in the Discussion. 

Minor comments regarding clarity:
1. The origin/existence of H3K27ac mark data are unclear and surprising when first mentioned on page 5, line 14 in an analysis. Consider introducing these data earlier.
2. The text referring to Figure 3B (page 5, line 39) describes 2.1% of the genome that switched compartments after exposure, but Figure 3B shows a region that did not change with exposure. Why show such a region? This figure could be removed. An example of altered TAD boundaries would be more valuable to allow readers to assess the magnitude of thee alternations in genome organization described in this section of the paper. 
3. Figure 4 would be more clear if the colors were consistent across panels. For example, TAD boundaries are red in A but green in B and C.
4. Footnotes or other descriptions for Additional file 9 should be provided. The referring text lists 3517 combinations; what do the ~22,000 rows represent? What is the meaning of the two 'filter' columns in Additional file 9?

**Reviewer 2**

Minor comments
1. the statement that GWAS signals tend to be located in regulatory regions is too often stated. Please rephrase.
2. Why was tumor necrosis factor-α (TNFα) choosen and not another inflammatory cytokine? There is no reference given to support this choice.
3. the paragraph about TAD boundaries (page 5/ line 5-23) is difficult to understand, please rephrase.
4. Page 6/ line 46: What were the reasons to choose AIDA for functional follow-up and not any of the other 15 SNP/Gene combinations?

**Authors’ response to reviewers**

**Reviewer #1**

***Lalonde, Codina-Fauteux and colleagues describe genomic data sets for endothelial cells as a resource for interpretation of genetic studies such as those for coronary artery disease. They generated RNA-seq, ATAC-seq, Hi-C and histone mark ChIP-seq data in resting and inflammation-stimulated aortic endothelial cells from a diploid immortalized cell line, teloHAEC and showed concordance with primary aortic endothelial cells. Integrative analyses linked thousands of regulatory elements to promoters and demonstrated that TNF treatment can lead to hundreds of differentially accessible chromatin sites corresponding to changes in gene expression.  CRISPR-based heterozygous deletion of one regulatory element show evidence that the element may affect expression of the AIDA gene.***

***The data sets created here provide new genomic characterization of endothelial cells in a resting versus a perturbed state. Such perturbation experiments are relatively new, and each example will interest a broad audience in genomics for the approaches and results of perturbation and integrative analyses.***

**Response.** We thank Reviewer #1 for the generally positive and extremely constructive comments. We also believe that our manuscript will be of broad interest to the genetic and functional genomic communities.

***Question 1. The authors use HiC data to connect regulatory elements to target genes.  The links would be more robust if the strength of ATAC-seq signals and RNA-seq expression levels were shown to be correlated (e.g. PMID 30858613).***

**Response.** We thank the Reviewer for this interesting suggestion. We note, however, that there is a fundamental difference between our analyses and the cited paper. In the article by Ulirsch et al., the authors have ATAC-seq and RNA-seq data from multiple donors and can therefore test whether a specific ATAC-seq peak signal is correlated with the expression of a gene linked by promoter Hi-C. The strength of this approach is that the correlation at each linked pair of ATAC peak – gene expression can be tested individually (across the multiple donors). This approach led the authors to prioritize >400 combinations, but did not test whether ATAC-seq peaks and the expression of genes linked by Hi-C are correlated when considering all possible combinations.

In our case, we only have data from 1 donor (teloHAEC) so we cannot test each combination of Hi-C-linked ATAC peak and gene expression level individually. However, we can test whether ATAC-seq peaks are correlated with the expression of the Hi-C-linked genes across all prioritized combinations. Although it is intuitive to think that if a regulatory element is strongly active (i.e. an open ATAC-seq peak), it should be linked to a gene that is highly expressed, this does not need to be the case. Indeed, transcriptional repressors could strongly open ATAC-seq peaks but result in weak or no gene expression. Furthermore, the prioritized ATAC-seq peaks were selected because they harbor genetic variants, and these variants could impact the relationship between ATAC-seq peak openness and gene expression levels.

With these caveats in mind, we set out to test the Reviewer’s suggestion on our own data. We selected combinations of ATAC-seq peaks and genes based on the original criteria of our paper: we required >10 reads to support Hi-C loops, removed weakly expressed genes (<10 percentile), and considered all timepoints (non-treated, 4hr TNFα, 24 hr TNFα). Across the 9,825 combinations of ATAC peaks and gene expression levels, we found a negative correlation that was marginally significant (Spearman’s *ρ*=-0.0237; P-value=0.019). We then performed a post-hoc analysis to focus the correlation test on high-quality combinations by requiring >50 Hi-C reads that link ATAC-seq peaks and the promoter of genes (other filters remained constant). Across these 4,104 high-quality combinations, we found a positive correlation between ATAC-seq peak openness and gene expression levels (Spearman’s *ρ*=0.0530; P-value=6.9x10^-4^). Although this result looks very interesting, we recognize the limited value of this post-hoc analysis. For this reason, and also given the limitations discussed above, we decided not to present these results in our manuscript. Instead, we opted to increase the stringency of the filters used to prioritize combinations of ATAC-seq peaks and gene promoters presented in **Additional file 11**. See our response to Questions #7 and #8 below.

***Question 2. The differential gene expression analyses are described in the text as (linear) absolute fold change, but the plots (Figure 1) and 'Additional file' data are shown in the more typical log fold change. Reporting the number of differentially expressed genes based on the linear threshold seems to over-state the magnitude of differences observed. The text units should match the units in figures and tables.***

**Response:** We agree with the Reviewer. We have changed “linear” fold-change in the text to log_10_ fold-change (LFC). The threshold for significant effect is now absolute LFC >0.3.

***Question 3. Figures 1A and 1C show differentially expressed genes and differentially accessible peaks at 4 hours, but the referring text (page 4, lines 18 and 36) reports larger numbers. Which time point comparisons were used to count differential genes and peaks (all of 0-4, 4-24, 0-24?) or was an integrative analysis used? Do the gene and peak differences between timepoints overlap appropriately to justify the choice to combine the timepoints? The manuscript would be more valuable if the rationale for those choices was also explained/justified.***

**Response.** We apologize for the confusion. Instead of focusing on specific timepoints for the differential RNAseq and ATACseq analyses, we used all timepoints and replicates in an analysis of deviance, as implemented in DESeq2 and described in the Methods section, to calculate statistical significance (P-values) and false discovery rate (FDR). We retained genes and ATACseq peaks with FDR<0.1%. We also filtered on effect sizes, requiring an absolute log10 fold-change >0.3 in any of the 3 comparisons (NT vs. 4hrs; NT vs. 24hrs; 4hrs vs. 24hrs). FDR and all LFC are readily available in **Additional files 2** and **4**. We have changed the main text and **Figure 1**’s legend in order to clarify our analyses.

RNA-seq section (page 4):

In total when considering all replicates and timepoints, we identified 1,316 differentially expressed genes (false discovery rate (FDR) <0.1% and absolute log_10_ fold-change (|LFC|) >0.3)(**Figure 1A** for the comparison of NT vs*.* 4 hours TNFα treatment, **Additional file 1** for all other comparisons, and **Additional file 2** for the complete list of differentially expressed genes).

ATAC-seq section (page 4):

By combining data from these different timepoints, we identified 95,491 ATACseq peaks, including 3,138 peaks (3.3%) that are differentially opened or closed (FDR <0.1% and |LFC|) >0.3) upon TNFα stimulation (**Figure 1C** for the comparison of the NT *vs.* 4 hours TNFα treatment, **Additional file 1** for all other comparisons, and **Additional file 4** for the complete list of differentially opened or closed ATACseq peaks).

Method section (page 12-13)

Genes with a false discovery rate (FDR, Benjamini & Hochberg correction) <0.1%, and log_10_ fold-change ≥0.3 or ≤-0.3 in any of the 3 possible comparisons (NT vs. 4 hours; NT *vs*. 24 hours; 4 hours *vs*. 24 hours) were considered differentially expressed.

ATACseq peaks with a false discovery rate (FDR, Benjamini & Hochberg correction) <0.1%, and log_10_ fold-change >0.3 or <-0.3 in any of the 3 possible comparisons (NT vs. 4 hours; NT *vs*. 24 hours; 4 hours *vs*. 24 hours) were considered differentially opened or closed.

Figure 1 legend (page 22)

(**A**) RNAseq of teloHAEC non-treated (NT) or treated with TNFα identified 1,316 differentially expressed (DE) genes (FDR <0.1% and absolute log_10_-fold-change >0.3) among three comparisons (NT vs. 4 hrs, NT vs. 24 hrs, 4 hrs vs. 24 hrs). Of these 1,316 genes, 836 genes were DE in the NT vs. 4 hrs comparison.

(**C**) ATACseq of teloHAEC NT or treated with TNFα identified 95,491 peaks, including 3,138 differentially opened (or closed)(DO) peaks (FDR <0.1% and absolute log_10_-fold-change >0.3) among three comparisons (NT vs. 4 hrs, NT vs. 24 hrs, 4 hrs vs. 24 hrs). Of these 3,138 peaks, 2,654 peaks were DO in the NT vs. 4hrs comparison.

For visualization, we can only represent comparison results for 2 timepoints, and we selected the NT vs. 4hrs TNFα treatment for **Figures 1A** and **1C**. We now provide the additional plots in **Additional file 1**.

***Question 4. The differential accessibility analyses (Figure 1C) appear to show an overall shift of peaks to more accessible after stimulation even among peaks considered not differentially accessible (shown in black), whereas the gene expression appears to be more equally increased versus decreased. Could the shift represent technical differences in ATAC-seq library preparation or analysis?  What quality control was performed to determine whether systematic technical differences exist between time points?  Alternatively, does the plot just appear to highlight the shifted peaks due to overlaying thousands of points, most of which are centered at log fold change of 0? If the latter, then the authors might consider showing these figures with density plots such as 'smoothScatter' in R ggplot2.***

**Response:** We do not think that the over-representation of ATAC-seq peaks in the non-treated vs. 4 hours TNFa treatment represent a technical or analytical artefact, but we think that the intuition from the Reviewer about the density of the data is correct. We created all ATAC-seq libraries using the same protocol and they were all sequenced at the McGill Genome Centre on the same sequencing lane. We used the same state-of-the-art analysis pipeline from ENCODE to process the reads and call peaks for all libraries, as detailed in the Methods. Using smoothScatter as suggested by the Reviewer, we can clearly see that most ATAC-seq peaks are centered at LFC=0, or slightly above. We added this figure in **Additional file 5**.

***Question 5. Methods and the threshold used to define linkage disequilibrium proxies of GWAS variants are missing.***

**Response.** We have added this information in the Methods section: “For the enrichment analyses of CAD, BP and BMI SNPs in open chromatin regions, we retrieved sentinel variants from published large-scale GWAS. We identified proxy variants in linkage disequilibrium (*r*^2^ >0.8) using populations of European ancestry from the 1000 Genomes Project.”

***Question 6. Figures 4B and 4C show an unusual wavy pattern of position of CAD sentinel SNPs relative to TAD boundaries. The waviness suggests that the results are driven by a very small number of loci or a non-random selection of 'matched' SNPs, affecting the conclusion of enrichment near TAD boundaries. The basis for the waves and impact on the conclusions needs to be described further or the conclusions tempered.***

**Response:** We have highlighted this limitation and tempered our conclusion by adding the following text to the manuscript: **“**Because of the relatively small number of CAD and BP sentinels SNPs (175 and 357 variants, respectively), the distributions of their position relative to the TAD boundaries were uneven (**Figure 4B-C** and **Additional file 10**). For both CAD and BP, associated SNPs tended to be closer from the nearest TAD boundary than matched SNPs (median distance 75 kb for associated SNPs vs. 103 kb for matched SNPs, empirical P-values ≤0.04), although a larger number of sentinel variants is needed to provide a definitive answer to this question.”

***Question 7. Methods for the integrative analysis with eQTL data from GTEx need to be described.  Are the reported links to gene using these eQTL data based only on a nominally significant p-value in GTEx or are these loci for which the GWAS and eQTL SNPs have been determined to be colocalized?  GTEx has reported that most GWAS variants that show nominal association with expression level of a gene are not colocalized, so most of these 3517 links to genes may be false positives. Here, eQTLs were just used to prioritize among the presumably many more links based on HiC data, so the set can still be used, but the limitation should be noted.  The relationship between CAD SNPs and the eQTL at AIDA should be described more explicitly.***

**Response:** The reviewer is correct: we initially used nominal GTEx P-value <0.05 to promote interesting eQTLs, and indeed many of these associations might be false positives. It is important to add, as pointed out by the Reviewer, that we used the GTEx data to support combinations of SNPs, ATACs-seq peaks, and genes that we had identified using our independent Hi-C data. In this context, and given that even GTEx has limited power to identify weak eQTLs, it is difficult to determine what significance thresholds we should use. We decided to increase the stringency of the GTEx filter by selecting eQTLs with P-values <0.001 (although we acknowledge that this remains an arbitrary choice). We also completed the information about the eQTL at *AIDA* – the top candidate gene in our analysis – in the manuscript (P_eQTL_=1.4x10^-6^ in esophagus muscularis).

Given this question, as well as Questions #1, #8 and #16, we have revised the filters used to prioritize high-quality combinations of SNPs – ATAC-seq peaks – genes linked by Hi-C and eQTL evidence. In particular, we focused on Hi-C loops that are supported by at least 20 reads (instead of 10) and eQTL effects with a GTEx P-value <0.001 (instead of <0.05). This resulted in a list of 991 high-quality combinations, corresponding to 38 CAD and 92 loci. We have edited the abstract and main text accordingly:

Abstract (p. 1)

This analysis highlighted 991 combinations of regulatory elements-promoters that map to 38 CAD and 92 BP GWAS loci.

Results (p. 6)

After filtering, this analysis identified 991 combinations of open chromatin regions and genes linked by physical 3D interactions and eQTL results (**Additional file 11**). These combinations map to 38 CAD and 92 BP GWAS loci. The average physical distance between these regulatory elements and gene promoters is 154±158 kb (**Additional file 11**)

We have also added a thorough description of the in silico strategy used to combine and integrate the genetic and genomic data in **Additional file 11**.

***Question 8. Further, the strategy applied by the 'in house scripts' (page 15 line 40) used to combine and integrate SNP, ChIP-seq, ATAC-seq, HiC, RNA-seq and GTEx data need to be further described.***

**Response:** We fully agree, and note that this important point was also raised by the editor. We have added in **Additional file 11** a very detailed step-by-step description of the strategy used to prioritize high-quality combinations of GWAS SNPs – ATAC-seq peaks – genes.

***Question 9. The accounting of 'combinations' of regulatory elements and promoters would be more informative for validation purposes if it also counted the total number of GWAS loci at which these elements and genes were located.***

**Response:** We have added this information in the abstract and the description of results: “After filtering, this analysis identified 991 combinations of open chromatin regions and genes linked by physical 3D interactions and eQTL results (**Additional file 11**). These combinations map to 38 CAD and 92 BP GWAS loci.”

***Question 10. Evidence that the CRISPR clones were heterozygous should be provided.***

**Response:** We added **Additional file 12** with results from a PCR amplification at the *AIDA* locus using out-out PCR primers. Using this genomic DNA amplification strategy, we clearly show that the parental cell line and non-deleted clones have the expected 1,410-bp PCR product whereas the 3 heterozygous clones have both the wild-type (1,410-bp) and deleted (389-bp) alleles.

***Question 11. The results described and shown in Figure 5 should more clearly indicate that the 6 points are not independent replicates, as only three heterozygous clonal lines were generated. The methods should better explain how these experiments were performed; were replicate wells of cells stimulated or were replicates performed at the qPCR stage?  The independence of the points influences whether the statistics based on 6 points are fair. Given the relatively large variance observed in these assays, the independence of values is especially important and affects conclusions in the Results and Discussion ('could not be induced' page 8 line 30).***

**Response:** To generate results in Fig. 5 B-C, we performed two independent experiments using 3 independent clones for the non-deleted controls and three independent clones that are heterozygous for the deletion. We have re-drawn the figures by color-coding in blue and cyan the 2 different batches. We have also edited the figure legend: ”Relative *AIDA* expression in teloHAEC without or with TNFα treatment for 4 hours with qPCR assay #1. Data was obtained from two independent experiments (circles in blue and cyan indicate the different biological replicates). For the non-deleted (Non-Del) and CRISPR/*Cas*9 heterozygote (Del) data points, we pooled data from three independent clones. Mean and standard deviation are plotted (black). For statistical analysis, we used linear regression correcting for batch effects, and report two-tailed *P*-values.”

Finally, we previously tested the statistical impact of batch effects on our results. Because they were non-significant, we originally used a simple t-test to assess significance. To fully address the Reviewer’s concern, we replaced the t-test by linear regression, correcting for batch effects. Conclusions remain unchanged.

***Question 12. The conclusion (Results, page 7, line 4) that the variant located within the element presumably affects expression of AIDA is speculative, as no data specific to the variant is shown. This speculation could be included in the Discussion.***

**Response:** We already discussed this limitation in the last paragraph of the Discussion. Specifically, we mentioned that two limitations of our study are that we failed to retrieve homozygous deleted clones and that we targeted a 1-kb region as opposed to a specific SNP. We have added the following text to clarify our discussion: “Although rs17163363 is the only variant in LD with the CAD sentinel variant rs67180937 within the CRISPR/Cas9 deletion generated at the *AIDA* locus, we cannot conclude that it is causal as other unknown variants in the deleted fragment could mediate the effect on *AIDA* expression. To address the potential causal role of rs17163363 in CAD, we propose that an allele replacement experiment, potentially mediated by CRISPR/Cas9 homology directed repair, is needed.”

***Question 13. The origin/existence of H3K27ac mark data are unclear and surprising when first mentioned on page 5, line 14 in an analysis. Consider introducing these data earlier.***

**Response:** We added the following sentences in the same paragraph than our introduction of the ATACseq data (page 4): “To further characterize our ATACseq open chromatin dataset, we generated histone H3 lysine 27 acetylation (H3K27ac) data in NT and TNFα-treated teloHAEC using chromatin immunoprecipitation followed by sequencing (ChIPseq). H3K27ac marks highlight regions of active transcription, and are found at enhancers and promoters. Within each condition (NT or with TNFα), we found that 70-74% of the ATACseq peaks intersected with H3K27ac peaks.

***Question 14. The text referring to Figure 3B (page 5, line 39) describes 2.1% of the genome that switched compartments after exposure, but Figure 3B shows a region that did not change with exposure.  Why show such a region? This figure could be removed. An example of altered TAD boundaries would be more valuable to allow readers to assess the magnitude of thee alternations in genome organization described in this section of the paper.***

**Response:** We have replaced the data originally presented in **Figure 3B** by the inflammatory E-selectin (SELE) locus to illustrate a B-to-A compartment switch and the impact on gene expression and chromatin openness following exposure for 4 hours to TNFalpha. We have modified the figure legend accordingly.

***Question 15. Figure 4 would be more clear if the colors were consistent across panels.  For example, TAD boundaries are red in A but green in B and C.***

**Response:** We have modified the colors in **Figure 4** and **Additional file 10** so that TAD boundaries are all in the same color.

***Question 16. Footnotes or other descriptions for Additional file 9 should be provided.  The referring text lists 3517 combinations; what do the ~22,000 rows represent?  What is the meaning of the two 'filter' columns in Additional file 9?***

**Response:** In the first version of **Additional file 9**, we included ALL combinations of SNP, open chromatin region, and gene linked by HiC data, independent of additional filtering. This represented ~22,000 combinations, of which 3,517 combinations remained after applying filters. Retrospectively, we agree that a large number of these combinations are not convincing and that we should only provide combinations that sustain more stringent filters. For this reason, we have completely revised **Additional file 9** (now **Additional file 11** in the revised version of our manuscript), which now include 991 combinations of SNPs, ATAC-seq peaks, and genes. See our response to Questions #7 and #8 above. In **Additional file 11**, we also provide a very detailed description of the protocol used to generate and filter this list.

**Reviewer #2**

***Question 17. the statement that GWAS signals tend to be located in regulatory regions is too often stated. Please rephrase.***

**Response:** We assume here that the Reviewer means that the regulatory potential of these elements has not been confirmed experimentally. We agree. We have re-phrased these statements in the text by replacing “regulatory” by “non-coding”.

Results (p. 5)

Most genetic variation associated with complex human traits by GWAS is in non-coding regions.

One outstanding challenge in gaining biological insights from GWAS discoveries is to connect variants located in non-coding elements with their target genes.

***Question 18. Why was tumor necrosis factor-α (TNFα) chosen and not another inflammatory cytokine? There is no reference given to support this choice.***

**Response:** We have explored how we can activate teloHAEC in different ways, and have also tested IL1beta and oxidized LDL (oxLDL). ILbeta generates a response almost identical to TNFalpha. In our hands, oxLDL is much more variable and not ideal to generate a comprehensive genomic dataset such as the one we created here. Thus, we opted for TNFalpha because it is widely used in the context of endothelial cell activation (we now cite a review to illustrate this point), it is physiologically relevant, and generates a very robust and reproducible inflammatory response. We have added the following sentence to the main text: “We selected TNFα to activate teloHAEC because it generates a very robust and reproducible inflammatory response.”.

***Question 19. the paragraph about TAD boundaries (page 5/ line 5-23) is difficult to understand, please rephrase.***

**Response:** We assume that Reviewer #2 refers to the paragraph that starts by: “Topologically associated domains (TADs) are defined by a high density of contacts…”. We edited this paragraph based on Question #6 from Reviewer #1. We have also added more background information to simplify interpretation of the data.

***Question 20. Page 6/ line 46: What were the reasons to choose AIDA for functional follow-up and not any of the other 15 SNP/Gene combinations?***

**Response:** Of the most promising combinations of SNP/ATAC peak/HiC loop/RNA-seq/GTEx combinations of results, AIDA was the only locus associated with CAD, the remaining being associated with BP. We added the following sentence to the main text: “We selected the *AIDA* locus for further functional characterization because it is associated with CAD; the *TRAF1* SNP is associated with BP (**Figure 5A**).”
